# Comparative Proteomic Profiling between Each of Two Consecutive Developmental Stages of the Solanum Fruit Fly, *Bactrocera latifrons* (Hendel)

**DOI:** 10.3390/ijms19071996

**Published:** 2018-07-09

**Authors:** Chiou Ling Chang, Scott M. Geib

**Affiliations:** USDA, Agricultural Research Service, Daniel K. Inouye U.S. Pacific Basin Agricultural Research Center, 64 Nowelo Street, Hilo, HI 96720, USA; scott.geib@ars.usda.gov

**Keywords:** *Bactrocera latifrons*, proteomic analysis, developmental biology, life cycle

## Abstract

The Solanum fruit fly, *Bactrocera latifrons* (Hendel), has a complex life cycle including multiple stages (egg, larva, pupa, and adult). Understanding the details of “what”, “when”, “where”, “why”, and “how” many hundred thousand proteins operate in this insect, interact, and express between each two consecutive developmental stages at molecular level not only can expand our knowledge, but also lead to the development of novel fruit fly control techniques. We tried to find what, when, and where in this study. Why and how will be presented in upcoming papers. We conducted a proteome profiling using 2-D gel electrophoresis and mass spectrometry. Samples of 3-day-old eggs, 1- and 10-day-old larvae, 1- and 10-day-old pupae, 1- and 9-day-old females and males of *B. latifrons* were used. A custom peptide database, derived from the *de novo B. latifrons* whole genome assembly was used for peptide identification. Differentially expressed proteins (DEPs) with significant fold expression and protein functions between two consecutive developmental stages were identified, annotated, described, and listed in gel images and/or charts. With this foundational information, we are not only providing valuable information, but also any impacts due to the biotic or abiotic environmental factors can be identified and manipulated, and lead to further research on gene editing and biomarker discovery.

## 1. Introduction

New tools for fruit fly control using gene editing will be an important supplement to current chemical treatments, sterile insect techniques, and biological control. To initiate gene editing research, specific target genes must be identified and validated. There were ways to identify and validate target genes/proteins using genomic and transcriptomic approach. Proteomics has expanded the research and development of targets in some insects such as *Drosophila*, mosquito, aphid, honey bee, silkworm, and *Helicoverpa armigera*, especially expression proteomics that has been used for identification and quantification or expression level of proteins of the cell, tissue, organism at different developmental stages or at different environmental conditions [1]. However, changes on the proteome of the Solanum fruit fly depending on the developmental stage has not yet been reported.

Tephritids such as the solanum fruit fly, *Bactrocera latifrons* (Hendel) are native to South and Southeast Asia and distributed among China, Taiwan, Malaysia, Thailand, Laos, India, Pakistan, Tanzania, Kenya, and Hawaii [2]. It infests Solanancease and Cucurbitaceae crops and has the potential to permanently establish itself, and compete, and/or coexist with other tephritid fruit fly species [3]. Morphological and ecological characteristics have been described [3,4,5]. There are four stages (egg, larva, pupa, and adult) in its life cycle (~48 days), 21 days from egg to adult; 2–3 days for the eggs to hatch, 8–9 days for larval development, adult emergence in 10 days, and pre-oviposition period is 10–11 days. While general biological data are available, information on protein expression, which may point to key target genes useful for genetic control programs, is lacking. Current proteomics technologies not only identify protein expression, but also post-translation modifications and interactions among different insect proteins. Future studies are needed to compare proteomic results across taxonomic groups and life stages, leading to advances in our understanding of insect proteomics [1].

Understanding basic fruit fly molecular biology at each developmental life stage in their life cycle to learn “what” these differentially expressed proteins within each developmental stage and between each two subsequent developmental stages are, and when and where they express, has been the most important element for improving the quality of mass-rearing fruit flies for Sterilization Insect Technique (SIT). This information can be used as foundational/baseline information to later manipulate or identify the causes/pathway from any biotic or abiotic influences to explain why and how they express using gene editing techniques. Here, we descriptively report on proteomic profiles through development between each two consecutive stages from an egg to an adult of the Solanum fruit fly using proteomic approach for the first time.

## 2. Results and Discussion

This report discusses all the subsequent stages—egg (E) to larva (L), larva to pupa (P), pupa to male (M), pupa to female (F), 1-day-old male and female, and 9-day-old male and female.

Solanum fruit flies undergo three stages of development—egg, larva, and pupa—before emerging as adults. Under natural field conditions, approximately 9–587 eggs are laid below the skin of the host fruit. Eggs hatch within 2–3 days, and the larvae feed for about 8.5 days before pupation. Pupation occurred in the soil for about 10.2 days. Adults occur all year around and females start oviposition at 6–17 days and continue laying eggs for as long as 117 days [2]. In our observations under laboratory conditions, eggs hatch after three days, larvae feed for about 10 days, and pupae take 12 days to develop into adults. In this study, we aimed to evaluate the protein changes in lab-reared flies between 3-day-old eggs (E3) and 1-day-old larvae (L1), 10-day-old larvae (L10) and 1-day-old pupae (P1), 10-day-old pupae (P10) and 1-day-old females (F1), and 10-d-old pupae (P10) and 1-day-old males (M1).

During development, insects undergo considerable cellular remodeling requiring the combined actions of thousands of proteins. In this study, the proteome profiling was evaluated based on the comparison between sequential stages. Generally speaking, the pupal stage expressed the most number of significantly differentially expressed proteins. While the pupal stage was most differentiated from other stages in terms of protein expression, the egg stage showed the least changes in protein expression compared with the others. The descriptions below are based on development between two consecutive stages:

### 2.1. Eggs (E3)–Larvae (L1)

**Figure 1:**
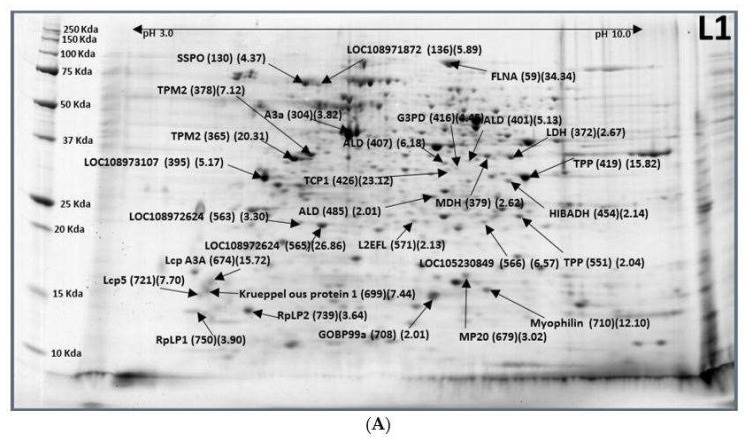

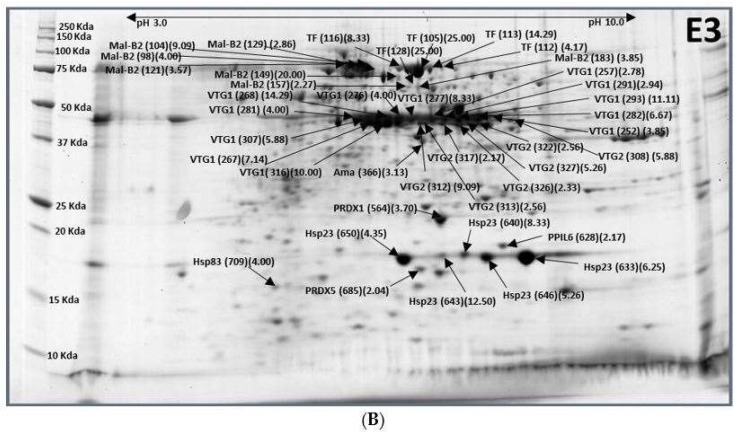
Representative images of 1-day-old larvae (**A**) and 3-day-old egg (**B**) of *Bactrocera latifrons*. Images were labelled with protein names (spot ID#) (L1/E3 ratios).

Protein changes in developmental process from eggs to larvae were the most significant changes of the stage. Between 3-day-old egg (E3) to newly hatched 1-day-old larvae (L1), there were a total of 70 significantly differentially expressed protein spots (Figure 1A,B). Among all, 41 protein spots containing 10 proteins were expressed as down-regulated by 51 to 96% (L1/E3 ratio = 0.04–0.49) (Figure 1B) whereas 29 protein spots containing 24 proteins were expressed as up-regulated by 2.01 to 34.34-fold (Table 1; Figure 1A)). 41 down-regulated protein spots were composed of 10 proteins including maltase 2 (7), transferrin (5), vitellogenin-1 (12), vitellogenin-2 (7), peroxiredoxin 1 (1), peroxiredoxin 5 (1), protein amalgam (1), peptidyl-prolyl cis-trans isomerase 6 (1), heat shock protein 23 (5), and heat shock protein 83 (1). Numbers inside parentheses following the protein names were the number of spots that belong to that same protein. Vitellogenin is the precursor of the lipoproteins and phosphoproteins that make up most of the protein content of yolk. At this moment, the embryogenesis is close to the end; therefore, both vitellogenin-1 and vitellogenin-2 were greatly downregulated by 64–93% and 54–89%, respectively. Heat shock protein 23 and 83 were decreased by 77–92%, and transferrin and maltase 2 were reduced by 76–96% and 56–95%, respectively. Protein amalgam (Ama) was downregulated 68%. Protein Ama (a secreted neuronal adhesion protein) contains three tandem immunoglobulin domains. It has both homophilic and heterophilic cell adhesion properties during early stages of *Drosophila* development and is required for axon guidance and fasciculation. The function of this protein remains elusive [6,7]. Peroxiredoxin 1 (Prx1) was downregulated by 73%. Peroxiredoxin 1 is involved in redox regulation of the cell. It is a molecular chaperone that acts as a regulator of hydrogen peroxide signaling. Downregulated Prx1 indicates the increase of survival (www.roswellpark.org/commercialization). It may play an important role in eliminating peroxides generated during metabolism by reducing peroxides through the thioredoxin system. It might participate in the signaling cascades of growth factors and tumor necrosis factor-alpha by regulating the intracellular concentrations of H_2_O_2_. It reduces an intramolecular disulfide bond in GDPD5 that gates the ability to GDPD5 to drive post-mitotic motor neuron differentiation. Ubiquitin-related modifier 1 (Urm1) is a ubiquitin-like molecule (UBL) with the dual capacity to act both as a sulphur carrier and posttranslational protein modifier. In the *Drosophila melanogaster*, Urm1 (CG33276) and its E1 activating enzyme Uba4 (CG13090) functioned together to induce protein urmylation in vivo. Urm1 conjugated to target proteins in general. Peroxiredoxin 5 (Prx5) is dependent on Uba4. A complete loss of Urm1 is lethal in flies, although a small number of adult zygotic Urm1 (n123) mutant escapers can be recovered. These escapers display a decreased general fitness and shortened lifespan, but they are resistant to oxidative stress. Therefore, Urm1 is a UBL that is involved in the regulation of JNK signaling and the response against oxidative stress in the fruit fly [8]. In this study, peroxiredoxin 5 (0.49) and Peptidyl-prolyl cis-trans isomerase 6 (0.46) did not show any significant changes between egg to larval stage. Heat shock protein 83 (hsp83) was downregulated by 75%. Hsp83 is a molecular chaperone that is involved in cell cycle control and signal transduction to promote the maturation, structural maintenance, and proper regulation of specific target proteins. Together with Hop (heat shot organizing protein) and piwi (regulatory proteins responsible for stem cell and germ cell differentiation), hsp83 mediates canalization, also known as developmental robustness. In *Ceratitis capitate,* Cchsp83 RNA expression is highly regulated during embryonic development, but the temporal fluctuations in RNA levels during embryogenesis were not followed by similar fluctuations in the levels of the protein [9]. Our results in *B. latifrons* confirmed this finding.

Twenty-four up-regulated proteins from 29 spots (Figure 1B) were SCO-spondin, Uncharacterized protein LOC108971872 isoform X2, Actin-2, muscle-specific, tropomyosin-2 (2), L-lactate dehydrogenase, malate dehydrogenase, Uncharacterized protein LOC108973107 isoform X1, fructose-bisphosphate aldolase (3), glyceraldehyde-3-phosphate dehydrogenase, trehalose-phosphate phosphatase (2), T-complex protein 1 subunit gamma, putative 3-hydroxyisobutyrate dehydrogenase MC, Uncharacterized protein LOC108972624 (2), Uncharacterized protein LOC105230849, protein lethal (2) essential for life, filamin-A, Larval cuticle protein A3A, muscle-specific protein 20, krueppelous protein 1, general odorant-binding protein 99a, myophilin, larval cuticle protein 5, 60S acidic ribosomal protein P2, and 60S acidic ribosomal protein P1 (Table 1). Again, abovementioned numbers inside parentheses following the protein names represented the number of spots that belong to that same protein. SCO-spondin is a large glycoprotein with a multi-domain organization that is secreted early by the developing central nervous system. It is a crucial embryonic cerebrospinal fluid (eCSF) factor that has been shown to regulate the balance between proliferation and differentiation of the brain neuroepithelial cells [10]. SCO-spondin was upregulated by 4.37-fold at 1-day-old larvae. Both *H. armigera* A3a and A3b genes are expressed during pupal development and in the brain of newly enclosed adults based on polymerase chain reaction and northern blot analysis [11]. Muscle-specific Actin-2 was upregulated by 3.82-fold in the larvae in this study, which added a piece of information that is expressed in the larvae stage. The function of tropomyosin-2 was dictated as a fundamental step of myogenesis prior to regulating contraction in the sarcomere [12]. Myogenesis is the formation of muscular tissue, particularly during embryonic development. Our result with tropomyosin-2 upregulated by 7–20-fold in L1 larval stage seems to confirm their report. Proteins that involve glycolysis such as lactate dehydrogenase, malate dehydrogenase, fructose-bisphosphate aldolase, glyceraldhyde-3-phosphate dehydrogenase, 3-hydrozyisobutyrate dehydrogenase, and trehalose-phosphate phosphatase were all expressed upregulated. Trehalose phosphatephosphatase (TPP) converts trehalose-6-phosphate to free trehalose. Trehalose can protect cells from desiccation, dehydration, heat, cold, and oxidation by decreasing protein denaturation through protein-trehalose interactions [13]. T-complex protein 1 subunit gamma (TCP-1) was found to interact with actin and tubulin proteins, suggesting that the complex may have a role in maintaining the structural dynamics of the cytoskeleton of parasites [14,15]. In this transformation, TCP-1 was increased by 23-fold to support the cytoskeleton. Protein lethal (2) essential for life [L(2)efl] is a small heat-shock (HS)-homologous gene, identified on the right arm of the second chromosome at locus 59F4,5. It acts as a regulator of lateral transverse muscle shapes in *Drosophila melanogaster*. It was expressed in embryonic, larval, and adult somatic and heart muscles during *Drosophila* development [16]. L(2)efl protein has demonstrated muscle-specific expression in late embryos and accumulates in a dotty pattern close to the muscle cell membrane. Our results confirmed their finding [17]. Filamin-A is a key component of a versatile signaling scaffold complex [18,19,20,21,22] that anchors various transmembrane proteins to the actin cytoskeleton and provides a scaffold for a wide range of cytoplasmic and nuclear signaling proteins, andwas increased by 34-fold during this transformation from eggs to larvae. Uncharacterized protein LOC108972624 was increased by 26.86-fold. Muscle-specific protein 20 (Mp20) that is not detected in the asynchronous oscillatory flight muscles in *D. melanogaster*, but is found in most, if not all, other muscles (the synchronous muscles) [23]. Mp20 was upregulated 3-fold in L1 stage. Krueppelous protein 1 Myophilin RNA is first expressed late in embryogenesis at stage 15 in *Drosophila melanogaster*. It confirmed our finding that at 1st day of larvae, myophilin was increased by 12-fold. We speculated that it is necessary for filament assembly in all muscles, and flightin for stability of flight muscle thick filaments in adult flies [24].

### 2.2. Larvae (10-Day-Old, L10)–Pupae (1-Day-Old, P1)

**Figure 2:**
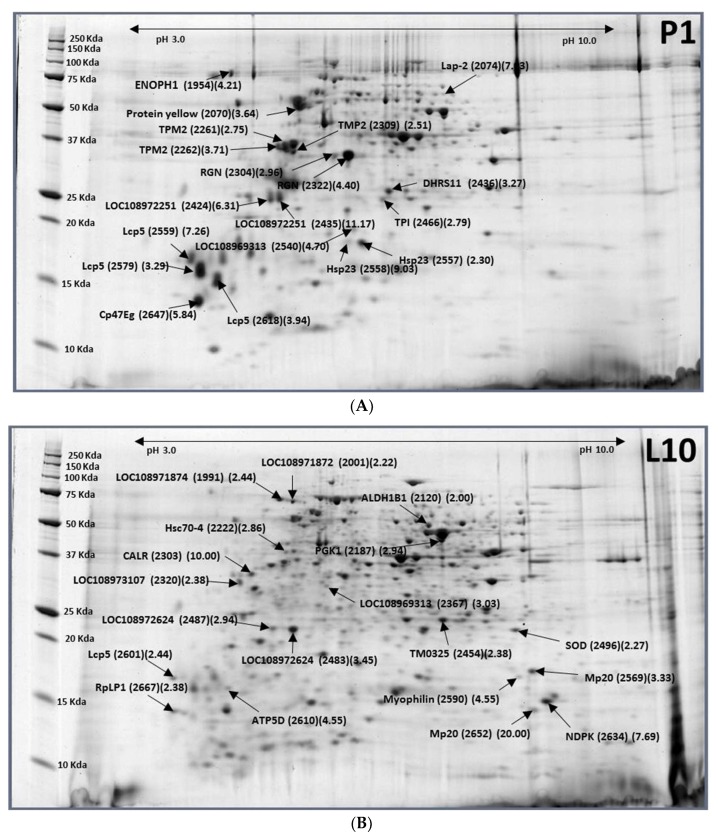
Representative images of 1-day-old pupae (P1) (**A**) and 10-day-old larvae (L10) (**B**) of *Bactrocera latifrons*. Images were labelled with protein names (spot ID#) (P1/L10 ratios).

Protein changes between last day of larvae (L10) and 1st day pupae (P1) were 19 protein spots containing 17 proteins expressing down-regulated by 50 to 95% (P1/L10 ratio = 0.05–0.50) (Figure 2A) whereas 19 protein spots contain only 12 proteins expressing up-regulated by 2.30 to 11.17-fold, mainly cuticle proteins (Table 2 and Figure 2B). Among these differentially expressed proteins, cuticle protein changes the most. Larval cuticle protein 5 (Lcp5) up-regulated by 3.29, 3.94, 7.26)-fold, cuticular protein 47Eg (CP47Eg) by 5.84-fold, uncharacterized protein LOC108972251 by 17.48 (6.31, 11.17)-fold, and LOC108969313 by 4.7-fold. Other downregulated uncharacterized proteins (including LOC108971874, LOC108971872, LOC108973107, 108969313, and LOC108972625) were down-regulated by 0.29–0.4, enolase-phosphatase E1 by 4.21, Tropomyosin-2 (TPM2) up-regulated by 8.97 (2.51, 2.75, 3.71)-fold while muscle-specific 20 (MP20) and myophilin down-regulated by 70–95%. Regucalcin (RGN) up-regulated by 10-fold. Calreticulin (CALR) was down-regulated by 90%. Protein yellow was upregulated by 3.64-fold in the transition from L10 to P1 stage. Protein yellow is only found in insects. In *Drosophila*, it is involved in pigmentation and male mating behavior, though the mechanism by which it influences melanization is not clear. SCO-spondin was down-regulated a little. In vitro, SCO-spondin was reported as a promotor of neuronal survival and differentiation, but its function in vivo is still unclear [10]. Heat shock protein 23 (hsp23) was up-regulated by 2.03 and 9.33-fold while heat shock protein cognate 4 (hsc4) was down-regulated by 65%. Heat shock proteins function as important chaperones intra-cellularly for other proteins in protein–protein interactions such as folding and assisting in the establishment of proper protein conformation (shape) and prevention of unwanted protein aggregation. The heat shock cognate genes play critical roles in chaperoning of cellular proteins, and they are expressed in a more constitutive manner compared to the inducible heat shock genes [25]. Aldehyde dehydrogenase X, phosphoglycerate kinase, oxidoreductase TM_0325, superoxide dismutase [Mn], and nucleoside diphosphate kinase were down-regulated by 50–87% while triosephosphate isomerase, dehydrogenase/reductase SDR family member 11, and aminopeptidase W07G4.4 were upregulated by 2.79–7.03 fold. 60S acidic ribosomal protein P1 was down-regulated by 58%.

### 2.3. Pupae (12-Day-Old, P12) to Females (1-Day-Old, F1)

**Figure 3:**
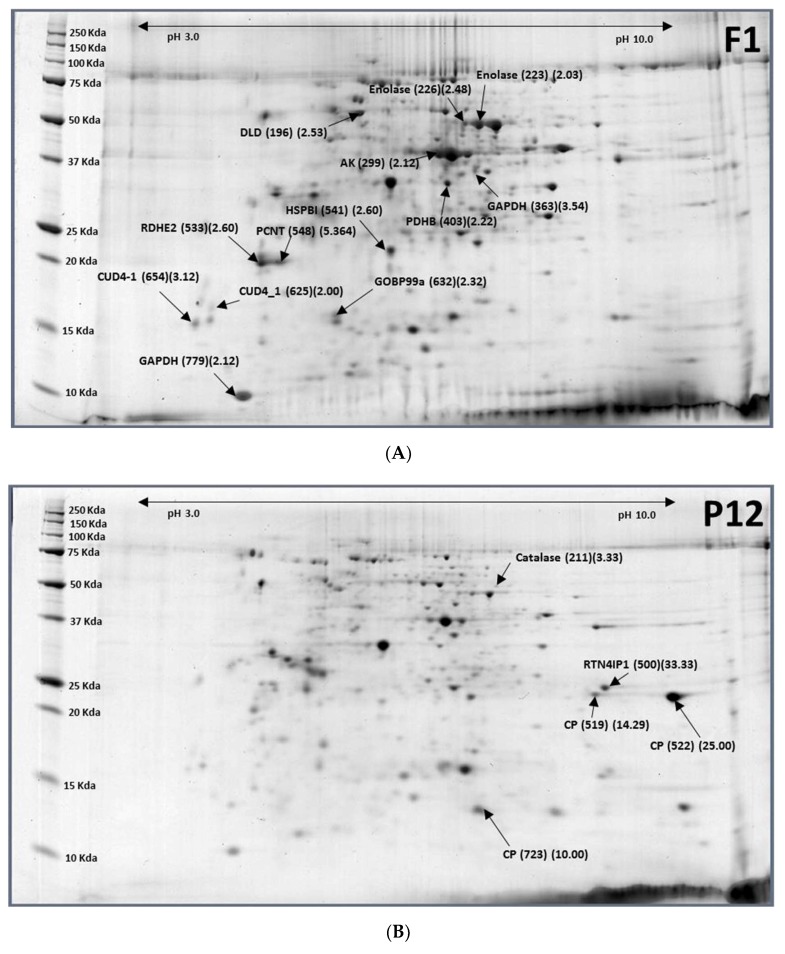
Representative images of 1-day-old females (F1) (**A**) and 12-day-old pupae (P12) (**B**) of *Bactrocera latifrons*. Images were labelled with protein names (spot ID#) (F1/P12 ratios).

Major differentially expressed proteins between 12-day-old pupae and 1-day-old females were 3 down-regulated proteins (5 spots)—Reticulon-4-interacting protein 1 (RTN4Ip1), cuticle protein (CP), catalase, and 13 up-regulated protein spots containing 10 proteins-Dihydrolipoyl dehydrogenase (DLD), enolase, arginine kinase (AK), glyceraldehyde-3-phosphate dehydrogenase (GAPDH), pyruvate dehydrogenase E1 component subunit beta (PDHB), epidermal retinol dehydrogenase 2 (RDHE2), heat shock protein beta-1 (HSPB1), pericentrin (PCNT), endocuticle structural glycoprotein ABD-4 (CUD4-1), and general odorant-binding protein 99a (Table 3; Figure 3). Reticulon-4-interacting protein 1 (RTN4Ip1) plays a role in the regulation of retinal ganglion cell (RGC) neurite outgrowth, and hence in the development of the inner retina and optic nerve or dendrite development. All proteins involved in glycolysis (DLD, AK, GAPDH, PDHB) were upregulated. Epidermal retinol dehydrogenase 2 (RDHE2) is involved in the pathway retinol metabolism, which is part of cofactor metabolism. Heat shock protein beta-1 (HSPB1) functions as a molecular chaperone. It maintains denatured proteins in a folding-competent state and plays a role in stress resistance and actin organization. It may regulate numerous biological processes through its molecular chaperone activity including the phosphorylation and the axonal transport of neurofilament proteins. Pericentrin is an integral component of the centrosome. The centrosome serves as a multifunctional scaffold for anchoring numerous proteins and protein complexes. Endocuticle structural glycoprotein ABD-4 (CUD4_1) plays a role in cell–cell interaction.

### 2.4. Pupae (12-Day-Old, P12) to Males (1-Day-Old, M1)

**Figure 4:**
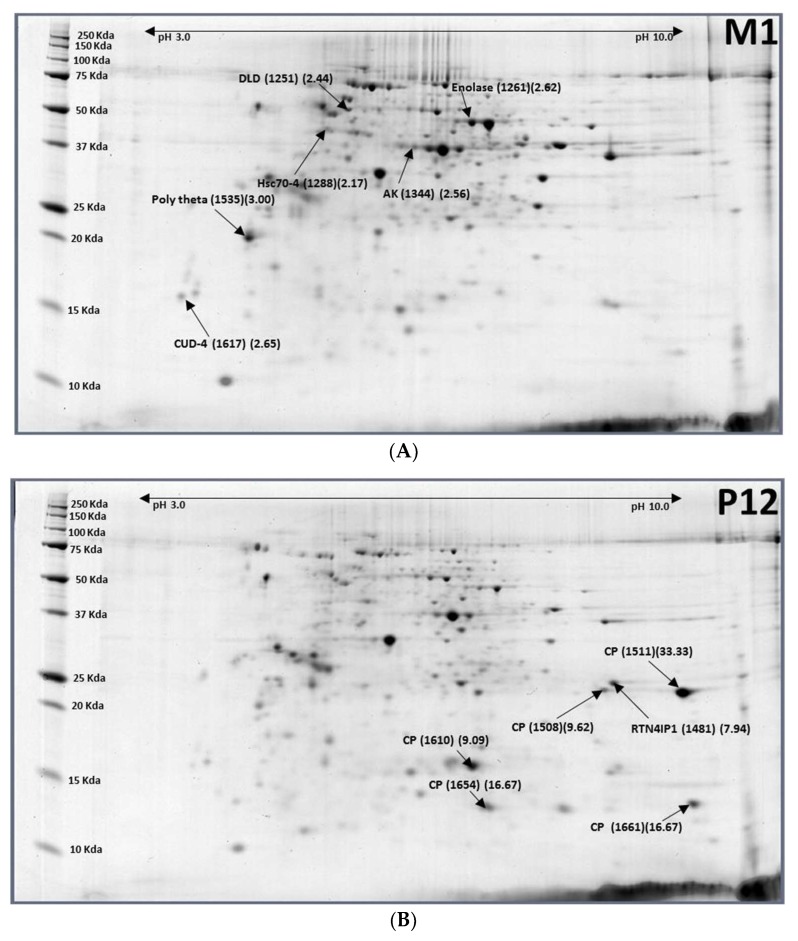
Representative images of 1-day-old males (**A**) and 12-day-old pupae (**B**) of *Bactrocera latifrons*. Images were labelled with protein names (spot ID#) (M1/P12 ratios).

Major differentially expressed protein between 12-day-old pupae and 1-day-old males were six down-regulated protein spots containing four cuticle proteins and six up-regulated protein spots including six proteins-DLD, enolase, Heat shock protein cognate four (Hsc70-4), Arginine kinase (AK), DNA polymerase theta (Pol theta), and endocuticle structural glycoprotein ABD-4 (Table 4; Figure 4). Cuticle proteins were reduced by 89–97% (M1/P12 ratio = 0.03–0.11). Glycolysis proteins such as enolase, DLD, and AK were upregulated to restore from hibernated pupae to adult stage. Enolase (phosphopyruvate hydratase) is a metalloenzyme responsible for the catalysis of the conversion of 2-phosphoglycerate (2-PG) to phosphoenolpyruvate (PEP) in the ninth and penultimate step of glycolysis. AK can maintain ATP levels by the phosphorylation of the so-called “phosphagens”, to rapidly replenish ATP. The function of Hsc70-4 is chaperone-binding/unfold protein-binding. Overexpression of DNA polymerase theta (Pol theta) in *Drosophila melanogaster* causes reduced hatch rate, and sensitivity to nitrogen mustard was reported. Polymerase theta (Pol theta) levels are important for genomic stability.

From pupal stage to adults, five proteins were identical between males and females. They were cuticle proteins (XP_018788732.1), dihydrolipoyl dehydrogenase (XP_018803223.1), enolase (XP_018794316.1), arginine kinase (XP_018785511.1), and endocuticle structural glycoprotein (XP_004520585.1). However, the differentially expressed proteins in females are more than in males.

### 2.5. Females (1-Day-Old)–Males (1-Day-Old)

**Figure 5:**
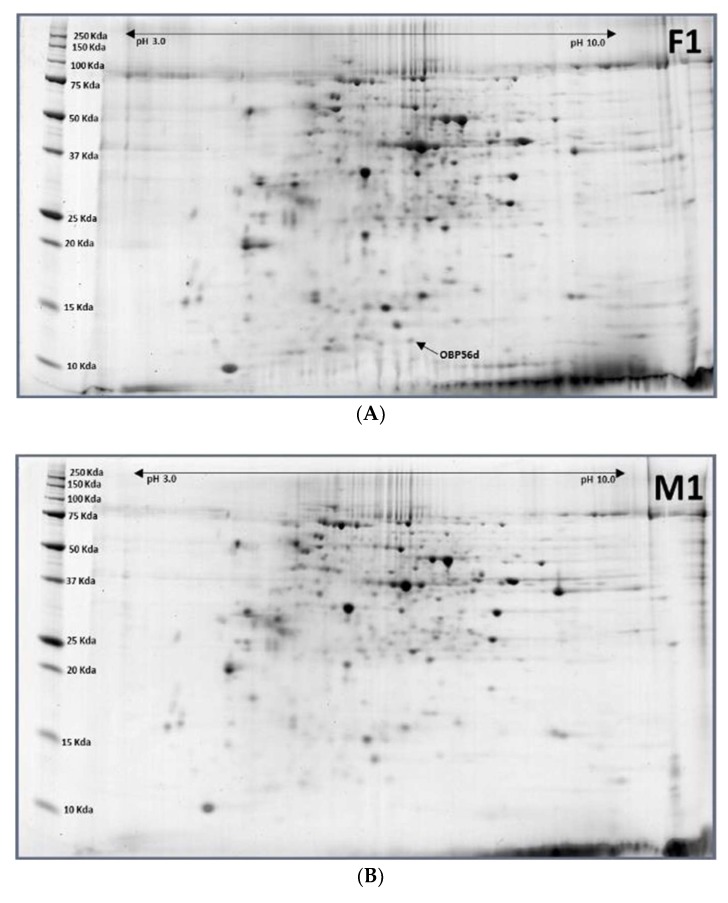
Representative images of 1-day-old females (F1) (**A**) and 1-day-old males (M1). (**B**) of *Bactrocera latifrons.* Images were labelled with protein names (spot ID#) (F1/M1 ratios).

Only one protein spot expressed significantly differently between 1-day-old females and males (Figure 5). That was OBP56d. The same protein was expressed at pupal stage [26].

### 2.6. Females (9-Day-Old)–Males (9-Day-Old)

**Figure 6:**
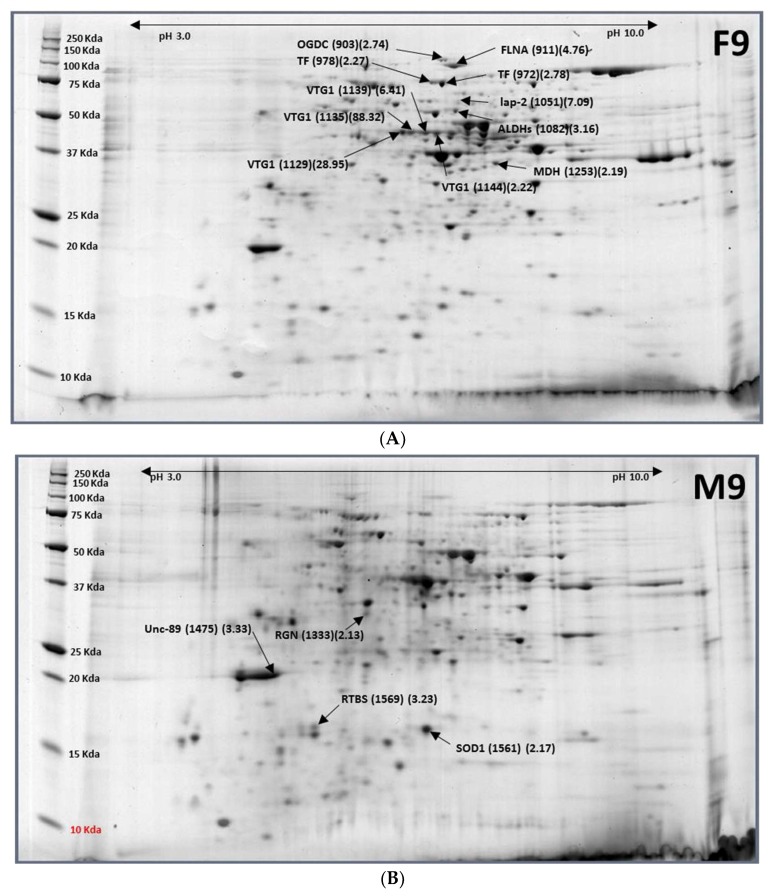
Representative images of 9-day-old females (**A**) and 9-day-old males (**B**) of *Bactrocera latifrons*. Images were labelled with protein names (spot ID#) (F9/M9 ratios).

There were 15 differentially expressed protein spots between nine-day-old females and males. Four down-regulated protein spots contained four proteins, and 11 up-regulated protein spots contained eight proteins (Table 5 and Figure 6). Females significantly and differentially expressed vitellogenin-1 by >120 fold over males.

## 3. Materials and Methods

We established a proteomic profile between sequential developmental stages of the solanum fruit fly using 2D gel electrophoresis coupled with mass spectrometry for protein identification. Database searches were performed with Matrix Science’s Mascot search engine v. 2.4 (www.matrixscience.com) on an in-house server against a concatenation of NCBI nr Insecta database, combined with a *B. latifrons* species-specific peptide database, derived from NCBI TSA BioProject: PRJNA281765. Differentially expressed proteins were compared and identified between each of two consecutive developmental stages and putative protein functions were annotated.

### 3.1. Insects

The *B. latifrons* colony collection was obtained initially from the USDA-ARS rearing facility in Hilo, HI, USA. Samples of varying ages of eggs (3 days), larvae (1 and 10 days), pupae (1 and 12 days), males (1 and 9 days), and females (1 and 9 days) were appropriately collected and stored at 80°C for protein analysis process.

### 3.2. Protein Extraction

We followed our standard proteomic protocols for sample preparation, 2D electrophoresis, and MS/MS (mass spectrometry) analysis [26]. Samples from all stage samples (0.25 g adults or pupae, 0.7 g eggs or larvae/mL buffer), were homogenized in one mL 10 mM Tris-HCl (pH 7.0) containing protease inhibitors (final dilution = 1:100; Sigma, St. Louis, MO, USA) for 15 s three times, with 30 s intervals using a Fast Prep-24 Instrument (MP Biomedicals, Solon, OH, USA). Homogenates were centrifuged twice at 15,294g for 15 mins at 4°C. The resulting infranatants were transferred to new vials on ice for immediate use.

### 3.3. Protein Quantification

Total proteins were quantified using the Pierce Micro BCA Protein Assay Kit, using BSA as a quantitative standard (Thermo Scientific, Rockford, IL, USA). Three independent biological replicates from the same generation were processed for each treatment.

### 3.4. 2D-Electrophoresis

#### 3.4.1. IEF (Isoelectric Focusing): Separating Different Proteins Based on pH Value

Five microliters of 2D gel protein standards (Bio-Rad, Hercules, CA, USA, #161-0320) were added to each sample tube. IPG strips (pH 3–10; 11 cm; Bio-Rad, #163-2014) were rehydrated overnight with these sample solutions. Iso-electric focusing was performed with a Protean IEF cell system (Bio-Rad, Hercules, CA, USA) using the standard protocol and a preset linear volt ramp program (8000 V and 50 μA/strip max., 35,000 vH). The focused strips were stored at −80 °C for later use.

#### 3.4.2. SDS Gel Electrophoresis Based on Molecular Weight (MW)

The IPG strips were equilibrated (15 min/buffer: 6 M urea, 2% SDS, 20% glycerol, 130 mM DTT, 0.375 M Tris-HCl, pH 8.7 [Buffer I] followed by 6 M urea, 2% SDS, 20% glycerol, 135 mM iodoacetamide, 0.375 M Tris-HCl, and pH 8.7 [Buffer II]) for the second dimension. Prior to running, molecular weight standards were applied (10 μL/lane, Bio-Rad #161-applied to each gel (precast gels, 8–16% Tris-HCl, Bio-Rad #345-0105), and proteins were separated on SDS-PAGE using the Criterion Cell system (Bio-Rad, Hercules, CA, USA, #165-6001). Gels were stained with Coomassie Blue G-250 (BioSafe Stain, Bio-Rad, Hercules, CA, USA) and scanned using densitometer (Bio-Rad GS-900), and the images were analyzed using Delta 2D software (Decodon GmbH, Greifswald, Germany). Protein spots with ratios (either larger than two or smaller than 0.5) significantly different between treatments at 95% or above significant level using Students’ *t*-test, were removed from the gels using a 1.5 mm spot picker (The Gel Company, San Francisco, CA, USA) and stored at −80 °C trypsin digestion and MS/MS analysis. Three independent biological replicates were performed.

### 3.5. MS/MS Analysis: Identification of Protein Identity

In preparation for MS/MS analysis, proteins were digested with trypsin, extracted, and then lyophilized and reconstituted with water [27]. A portion of each protein was mixed with alpha-cyano-4-hydroxycinnamic acid matrix and applied to the MALDI target and analyzed. The resulting sequence data (ratios, standard errors, *p* values), combined with observed protein name, MW and pI values, protein score, ion score, protein %, E value, and peptide sequence (with highest ion score) were used to establish protein identities (Supplementary file 1).

### 3.6. Data Search and Analysis

Database searches were performed with Matrix Science’s Mascot search engine v. 2.4 (www.matrixscience.com) on an in-house server against the updated NCBInr “Blat 20160224” database and the NCBInr Insects database. The custom *B. latifrons* peptide database, generated from a draft annotation set of the *B. latifrons* genome is available as supplemental information (Supplementary files 2 and 3, NCBI BioProject 281765).

## 4. Conclusions

In summary, we found: 10 downregulated and 24 upregulated proteins between eggs and larvae, 17 downregulated and 12 upregulated proteins between larvae and pupae, 3 downregulated and 10 upregulated proteins between pupae and females, 4 downregulated and six upregulated proteins between pupae and males, and one was identified as significantly differentially expressed between 1-day-old females and males, while 4 downregulated and 8 upregulated proteins were identified between 9-day-old females and males. Proteins that expressed significantly differentially in both L1/E3 and P1/L10 are SCO-spondin (SSPO), hypothetical protein LOC108971872 isoform X2, LOC108973107 isoform X1, LOC108972624, muscle-specific protein 20 (msp20), myophilin, larval cuticle protein 5, 60S acidic ribosomal protein P1, and tropomyosin-2. Glyceraldeyde-3-phosphate dehydrogenase is the only protein differentially expressed at both L1/E3 and F1/P12. Heat shock protein cognate 4 is the only protein differentially expressed at both P1/L10 and F1/P12. At both F1/P12 and M1/P12, 5 proteins (cuticle protein, dihydrolipoyl dehydrogenase, enolase, arginine kinase, and endocuticle structural glycoprotein ABD-4 (CUD4_1) are differentially expressed. Actin has been reported by in *Ceratitis capitata*, the “Mediterranean fruit fly” or “Medfly” [1]. They found that this gene was presented in the Medfly during late larval and late pupal development as well as in thoracic and leg tissue preparations from newly emerged adults. However, we found the Actin gene significantly differentially expressed in the early stage of *B. latifrons* larvae. The pupal stage is the stage that produced the most significantly differentially expressed proteins.

Understanding fruit fly basic biology in the development of each life stage in their life cycle at molecular level has been the most important element for improving the quality of mass-rearing fruit flies for SIT. We have profiled the proteome analysis of *Bactrocera latifrons* development via proteomics approach. From this study, we learned “what” these differentially expressed proteins are within each developmental stage and between each of two subsequent developmental stages, and when and where they express. This information will be used as a foundational/baseline information to later manipulate or identify the causes/pathway from any biotic or abiotic influences to explain why and how they express using gene editing techniques. This study will supply a wealth of additional information on understanding fruit fly development at molecular level. Profiling all four fruit fly species in Hawaii, which represents three important genera, has been ongoing.

## Figures and Tables

**Table 1:** Differentially expressed proteins between 3-day-old eggs and 1-day-old larvae of *B. latifrons.*

Accession #	Protein Names (Abbreviations)	Spot ID (L1/E3 Ratio)
XP_018793231.1	Maltase 2 (Mal-B2)	E3-98 (0.25), 104 (0.11), 121 (0.28), 129 (0.35), 149 (0.05), 157 (0.44), 183 (0.26)
XP_018784870.1	Transferrin (TF)	E3-105 (0.04), 112 (0.24), 113 (0.07), 116 (0.12), 128 (0.04)
XP_018798053.1	Vitellogenin-1 (VTG1)	E3-252 (0.26), 257 (0.36), 282 (0.15), 291 (0.34), 293 (0.09)
XP_018787380.1	Vitellogenin-1 (VTG1)	E3-267 (0.14), 268 (0.07), 276 (0.25), 277 (0.12), 281 (0.25), 307 (0.17), 316 (0.10)
XP_018787290.1	Vitellogenin-2 (VTG2)	E3-308 (0.17), 312 (0.11), 313 (0.39), 317 (0.46), 322 (0.39), 326 (0.43), 327 (0.19)
XP_018791497.1	Protein amalgam (Ama)	E3-366 (0.32)
XP_011201883.1	Peroxiredoxin 1 (PRDX1)	E3-564 (0.27)
XP_018795769.1	Peptidyl-prolyl cis-trans isomerase 6 (PPIL6)	E3-628 (0.46)
XP_018788060.1	Heat shock protein 23 (Hsp23)	E3-633 (0.16), 640 (0.12), 643 (0.08), 646 (0.19), 650 (0.23)
XP_018797702.1	Peroxiredoxin-5, MC (PRDX5)	E3-685 (0.49)
XP_018799095.1	Heat shock protein 83 (Hsp83)	E3-709 (0.25)
XP_018793742.1	SCO-spondin (SSPO)	L1-130 (4.37)
XP_018793741.1	Uncharacterized protein LOC108971872 isoform X2	L1-136 (5.89)
XP_004526477.1	Actin-2, muscle specific	L1-304 (3.82)
XP_018799378.1	Tropomyosin-2 (TPM2)	L1-365 (20.31), L-378 (7.12)
XP_018802321.1	L-lactate dehydrogenase (LDH)	L1-372 (2.67)
XP_018783435.1	Malate dehydrogenase, cytoplasmic (MDH)	L1-379 (2.62)
XP_018795679.1	Uncharacterized protein LOC108973107 isoform X1	L1-395 (5.17)
XP_018789491.1	UDP-glucose 4-epimerase	L1-401 (5.13), 407 (6.18), 485 (2.01)
XP_018787334.1	Glyceraldehyde-3-phosphate dehydrogenase (G3PD)	L1-416 (4.45)
XP_018804543.1	Trehalose-phosphate phosphatase (TPP)	L1-419 (15.82)
XP_004519608.1	T-complex protein 1 subunit gamma (TCP1)	L1-426 (23.12)
XP_018804039.1	3-hydroxyisobutyrate dehydrogenase (HIBADH)	L1-454 (2.14)
XP_018804543.1	Trehalose-phosphate phosphatase B(TPP)	L1-551 (2.04)
XP_018794827.1	Uncharacterized protein LOC108972624	L1-563 (3.30), 565 (26.86)
XP_011210145.1	Uncharacterized protein LOC105230849	L1-566 (6.57)
XP_018793665.1	Protein lethal (2) essential for life (L2EFL)	L1-571 (2.13)
XP_018791794.1	Filamin-A, isoform X2 (FLNA)	L1-59 (34.34)
XP_018787727.1	Hypothetical protein c0_g1_i1 (c0_g1_i1)	L1-674 (15.72)
XP_018804351.1	Muscle-specific protein 20 (MP20)	L1-679 (3.02)
gi|880832606|	Krueppel ous protein 1	L1-699 (7.44)
XP_018799091.1	General odorant-binding protein 99a (GOBP99a)	L1-708 (2.01)
XP_018788026.1	Myophilin	L1-710 (12.10)
XP_014094514.1	Larval cuticle protein 5 (LCP5)	L1-721 (7.70)
XP_018797071.1	60S acidic ribosomal protein P2 (RpLP2)	L1-739 (3.64)
XP_018803514.1	60S acidic ribosomal protein P1 (RpLP1)	L1-750 (3.90)

**Table 2:** Differentially expressed proteins between 10-day-old larvae and 1-day-old pupae of *Bactrocera latifrons*.

Accession No.	Protein Names (Abbreviations)	Spot ID (P1/L10 Ratio)
XP_018793742.1	Uncharacterized protein LOC108971874	L10-1991 (0.41)
XP_018793741.1	Uncharacterized protein LOC108971872 isoform X2	L10-2001 (0.45)
XP_018783509.1	Aldehyde dehydrogenase X (ALDH1B1)	L10-2120 (0.50)
XP_018790778.1	Phosphoglycerate kinase (PGK1)	L10-2187 (0.34)
XP_011208284.1	Heat shock protein cognate 4 (Hsc70-4)	L10-2222 (0.35)
XP_018794703.1	Calreticulin (CALR)	L10-2303 (0.10)
XP_018795679.1	Uncharacterized protein LOC108973107 isoform X1	L10-2320 (0.42)
XP_018789492.1	Uncharacterized protein LOC108969313	L10-2367 (0.33)
XP_018802393.1	putative oxidoreductase TM_0325 (TM0325)	L10-2454 (0.42)
XP_018794827.1	Uncharacterized protein LOC108972625	L10-2483 (0.29), L10-2487 (0.34)
XP_018788990.1	Superoxide dismutase [Mn] (SOD)	L10-2496 (0.44)
XP_018804351.1	Muscle-specific protein 20 (Mp20)	L10-2569 (0.30), L10-2652 (0.05)
XP_018788026.1	Myophilin	L10-2590 (0.22)
gi|880854006|	Larval cuticle protein 5 (Lcp5)	L10-2601 (0.41)
XP_018804878.1	ATP synthase subunit delta (ATP5D)	L10-2610 (0.22)
XP_004517938.2	Nucleoside diphosphate kinase (NDPK)	L10-2634 (0.13)
XP_018803514.1	60S acidic ribosomal protein P1 (RpLP1)	L10-2667(0.42)
XP_018800124.1	Enolase-phosphatase E1	P1-1954 (4.21)
XP_018789027.1	Protein yellow	P1-2070 (3.64)
XP_018802734.1	Putative aminopeptidase W07G4.4 (lap-2)	P1-2074 (7.03)
XP_018799378.1	Tropomyosin-2 (TPM2)	P1-2261 (2.75), P1-2262 (3.71)
XP_018802440.1	Regucalcin (RGN)	P1-2304 (2.96), P1-2322 (4.40)
XP_018799300.1	Tropomyosin-2 (TPM2)	P1-2309 (2.51)
XP_018794340.1	Uncharacterized protein LOC108972251	P1-2424 (6.31), P1-2435 (11.17)
XP_018786140.1	Dehydrogenase/reductase SDR family member 11 (DHRS11)	P1-2436 (3.27)
XP_018793665.1	Triosephosphate isomerase (TPI)	P1-2466 (2.79)
XP_018789492.1	Uncharacterized protein LOC108969313	P1-2540 (4.70)
XP_018788064.1	Heat shock protein 23 (Hsp23)	P1-2557 (2.30), P1-2558 (9.03)
XP_018805087.1	Larval cuticle protein 5 (Lcp5)	P1-2559 (7.26)
XP_018793318.1	Larval cuticle protein 5 (Lcp5)	P1-2579 (3.29)
XP_018793320.1	Larval cuticle protein 5 (Lcp5)	P1-2618 (3.94)
XP_018805010.1	Cuticular protein 47Eg (Cp47Eg)	P1-2647 (5.84)

**Table 3:** Differentially expressed proteins between 1-day-old females (F1) (3A) and 12-day-old pupae (P12) (3B) of *Bactrocera latifrons.*

Accession No.	Protein Name	Spot ID (F1/P12)
XP_018798316.1	Catalase	P12-211 (0.30)
XP_018794196.1	Reticulon-4-interacting protein 1(RTN4lPI)	P12-500 (0.03)
XP_018794197.1	Cuticle protein (CP)	P12-519 (0.07)
XP_018794197.1	Larval cuticle protein A2B	P12-522 (0.04)
XP_018788732.1	Cuticle protein 8 (CP8)	P12-723 (0.10)
XP_018803223.1	Dihydrolipoyl dehydrogenase (DLD)	F1-196 (2.53)
XP_018794316.1	Enolase	F1-223 (2.03), F1-226 (2.48)
XP_018785511.1	Arginine kinase (AK)	F1-299 (2.12)
XP_018804575.1	Glyceraldehyde-3-phosphate dehydrogenase (GAPDH)	F1-363 (3.54)
XP_018798823.1	Pyruvate dehydrogenase E1 component subunit β (PDHB)	F1-403 (2.22)
XP_018801988.1	Epidermal retinol dehydrogenase 2 (RDHE2)	F1-533 (2.60)
XP_018800458.1	Heat shock protein beta-1 (HSPB1)	F1-541 (2.60)
XP_018801988.1	Pericentrin (PCNT)	F1-548 (5.64)
XP_004520585.1	Endocuticle structural glycoprotein ABD-4 (CUD-4)	F1-625 (2.00), F1-654 (3.12)
XP_018799095.1	General odorant-binding protein 99a-like (OBP99a)	F1-632 (2.32)
XP_018787334.1	Glyceraldehyde-3-phosphate dehydrogenase (GAPDH)	F1-779 (2.12)

**Table 4:** Differentially expressed proteins between 12-day-old pupae and 1-day-old males of *Bactrocera latifrons.*

Accession No.	Protein Name	Spot ID (M1/P12)
XP_018794197.1	Cuticle protein (CP)	P12-1511 (0.03)
XP_018783307.1	Cuticle protein (CP)	P12-1610 (0.11)
XP_018783299.1	Cuticle protein (CP)	P12-1654 (0.06)
XP_018788732.1	Cuticle protein 8 (CP8)	P12-1661 (0.06)
XP_018803223.1	Dihydrolipoyl dehydrogenase (DLD)	M1-1251 (2.44)
XP_018794316.1	Enolase	M1-1261 (2.62)
XP_014087797.1	Heat shock protein cognate 4 (Hsp70-4)	M1-1288 (2.17)
XP_018785511.1	Arginine kinase (AK)	M1-1344 (2.56)
XP_018801988.1	DNA polymerase θ (Poly theta)	M1-1535 (3.00)
XP_004520585.1	Endocuticle structural glycoprotein ABD-4 (CUD-4)	M1-1617 (2.65)

**Table 5:** Differentially expressed proteins between 9-day-old females and males of *B. latifrons.*

Accession No.	Protein Name	Spot ID (F9/M9 Ratio)
XP_018804032.1	Regucalcin (RGN)	M9-1333 (0.47)
XP_018801988.1	Muscle M-line assembly protein unc-89 (UNC-89)	M9-1475 (0.30)
NP_001291905.1	Superoxide dismutase [Cu-Zn] (SOD1)	M9-1561 (0.46)
XP_018799095.1	RNA-directed DNA polymerase from transposon BS (RTBS)	M9-1569 (0.31)
XP_018802735.1	Putative aminopeptidase W07G4.4 (LAP-2)	F9-1051 (7.09)
XP_018784524.1	Aldehyde dehydrogenase (ADH)	F9-1082 (3.16)
XP_018787380.1	Vitellogenin-1 (VTG1)	F9-1129 (28.95), F9-1135 (88.32),
		F9-1139 (6.41), F9-1144 (2.22)
XP_018783435.1	Malate dehydrogenase, cytoplasmic (MDH)	F9-1253 (2.19)
XP_018793355.1	2-oxoglutarate dehydrogenase (OGDC)	F9-903 (2.74)
XP_018791794.1	Filamin-A (FLNA)	F9-911 (4.76)
XP_018784870.1	Transferrin (TF)	F9-972 (2.78), F9-978 (2.27)

## Supplementary Materials

Supplementary materials can be found at http://www.mdpi.com/1422-0067/19/7/1996/s1.
