# Supplementary material for: Comparative Proteomic Profiling between Each of Two Consecutive Developmental Stages of the Solanum Fruit Fly, Bactrocera latifrons (Hendel)
Source: Int J Mol Sci. 2018 Jul 9;19(7):1996. doi: 10.3390/ijms19071996 (PMC6073878; doi:10.3390/ijms19071996)

Charles W. Gehrke Proteomics Center
Research Core Facilities
University of Missouri–Columbia
www.proteomics.missouri.edu

214 Bond Life Sciences Center
1201 East Rollins Road
Columbia, MO 65211
Dr. Brian Mooney, Assoc. Dir. (573) 884-7374
FAX (573) 884-9676
Mass Spectrometry (B. DaGue) (573) 882-5481

Analyst's Report

Sample Identification:

Date submitted: May 12, 2016
Submitted by: Dr. Cynthia L. Goodman (573-876-8303)
and Dr. Stella Chang, USDA-ARS, Hilo, Hawaii (808-959-4312)
P.I.: Dr. David Stanley, USDA, ARS, BCIRL, Research Park, Columbia, MO
MOCode: **DN778**
Sample(s):

112 trypsin digests of Coomassie Blue (G250)-stained 2-D gel plugs (pI range 3-10) were submitted on 5/12/2016. The samples were from *Bactrocera latifrons*.
MALDI TOF/TOF MS plus MS/MS analyses were requested.
The data for the samples were to be queried initially against the NCBInr *B. latifrons* protein database. **This database was downloaded from the NCBInr website and added to the old Mascot Server in the Proteomics Center on February 24, 2016.**

Comments Regarding Results:

The NCBInr *Bactrocera latifrons* database downloaded from NCBI on February 24, 2016, was incomplete at that time. A significant number of the sample digests contained peptides which provided high-quality MS/MS which were not assigned with statistically significant ion scores to proteins in the *B. lat.* database. Either *de novo* analysis of the poorly matched MS/MS spectra or searches against the NCBInr Insects database (as of 2/20/2015) showed the MS/MS to match more closely with proteins from other insects, in particular proteins from *B. dorsalis*. This was especially true for the digests of the high molecular weight proteins observed in "Egg." Transferrin was one of the high molecular weight proteins in "Egg" which was generally assigned a relatively low score most likely because the sequence for transferrin in the *B. lat.* database is only a partial sequence. By comparison, assignment of peptides to transferrin from *B. dor.* was much more convincing in the search *vs.* Insects. Unless the sample source was contaminated with *B. dor.* Egg proteins, these results suggest that the sequence of transferrin is well-conserved in these two Bactrocera species.

Intensely stained spots in a gel may leach protein during destaining of the gel. This leached protein can in turn contaminate nearby regions of the gel. As a consequence, digests of weak protein spots may show peptides derived from nearby intensely-stained protein spots. Furthermore, it is quite difficult to cleanly excise weak protein spots located adjacent to high abundance spots, and some cross-contamination would be expected.

For strings of protein spots differing by pI which are assigned to the same or similar proteins in a database search, it is generally very difficult to assess what is unique about each of the individual spots. In part this is the result of mixtures of modifications present in varying proportions in each of the gel spots in the string, with the relative amounts of some modifications being low. As an example, the five LAT E protein spots 633, 646, 640, 643, and 650 (listed from most basic to most acidic), were all assigned to one or both heat shock protein 23s (HSP23), gi| 880914899 or gi| 880898980 in *B. lat.* database searches. In order to account for more of the intense peptides seen in the digests of these proteins, the searches allowed the variable modifications Asparagine Deamidation,

and Acetylation of the protein N-terminus in addition to the usual Methionine oxidation and Pyroglutamate formation at peptide N-terminal Glutamine or peptide N-terminal Glutamate. The higher scores were for gi| 880914899 (theor. pI 7.11) for all but spot 650. Examination of the MS spectra for these samples showed that as one progressed from the most basic to the most acidic spot, the relative amounts of oxidation and deamidation of the HSP23 gi| 880914899 increased. Conversion of Asparagine to Aspartic Acid would indeed make the protein more acidic. Peptides corresponding to the N-terminal acetylated forms of both of the proteins were seen in the spectra and would be expected to result in a more acidic protein relative to the non-acetylated protein. (However, the expected non-acetylated N-terminal peptides were not clearly observed in any of these spot digests.) In addition, the amounts of the second HSP23, gi| 880898980 (theor. pI 5.96), were seen to increase as the pI of the spots decreased, with spot 650 containing essentially only HSP23 gi| 880898980. However, the modification which resulted in the pI difference between the two spots 643 and 650 containing HSP23 gi| 880898980 was not at all evident from the MS spectra. Thus, the picture is complex. Often recovery or ionization efficiency of the modified peptides is not sufficient to aid in the assessment of the changes occurring in a given protein as a function of the treatment of the organism. Furthermore, methionine oxidation and asparagine deamidation are among those peptide modifications which are often considered to be artifacts of sample preparation, sample storage, or the alkaline conditions used for trypsin digestion. (See attachment 1 at the end of the report.)

In the course of cross-checking spectra and database search results, an interesting comparison was seen between the larval proteins LAT L-ID2487 and LAT L-ID2483. Both appeared to be about 20 KDa, and they were near each other in the gel (image 3). LAT L-ID2487 was the less abundant and more acidic protein of the two. While the MS spectra for both samples contained ions which could be assigned to *B. lat.* "Hypothetical protein c0_g2_i1" (gi|880815061), there was one especially intense peptide ion at *m/z* 2261.15 Da which appeared ONLY in the MS of LAT L-ID2487. *De novo* sequence analysis of the MS/MS spectrum for *m/z* 2261.15 combined with a query against the NCBInr Insects database (2/20/2015), indicated with confidence that this peptide (present in several *B. dor.* proteins including gi|751804194) differed by a single amino acid (Proline substituted for Serine) from the major tryptic peptide at *m/z* 2251.14 seen in LAT L-ID2483 that was assigned to gi|880815061. The triplet codons for Proline are CCN while those for Serine are UCN and AGU/C. A BlastP alignment of the sequences for gi|880815061 and gi|751804194 showed 95% identity between the two proteins. One question raised by these results is whether or not the major protein component in LAT L-ID2487 is a *B. lat.* variant (which is not in the *B. lat.* database at this time) of the major protein component of LAT L-ID2483. OR is it possible that the LAT L-ID2487 protein is present as a consequence of the presence of *B. dor.* insect proteins in the source material thought to be purely from *B. lat.*? (See attachment 2 at the end of the report.)

The Investigators are encouraged to cross-check the precursor mass lists for each sample shown in the Spot Set tables against the database search results to see how many and which precursor ions were matched with better scores to proteins from insects other than *B. lat.* Any sample for which the database search results were inconsistent with the intensity of the Coomassie Blue staining should be evaluated in terms of the intensity of the ions in the MS spectra (which can be provided as needed), the quality of the MS/MS spectra (which can also be provided as needed), the number of MS/MS assigned, and the scores for the assigned MS/MS spectra from both database searches. The Analyst can provide assistance with this process upon request.

It must be pointed out that the protein coverages seen in the database search results Protein Summaries are probably somewhat inflated. The use of a mass error tolerance of ± 200 ppm for peptide masses and a signal-to-noise threshold of 20 for inclusion of ions in the MS spectrum peak list, will have resulted in some false positive assignments among ions matched on the basis of mass alone.

Work Summary and Notes:

In general, the day prior to analysis, the samples were lyophilized dry, the dry residues were reconstituted in 10 µL water, frozen, and dried again overnight. On the day following the overnight drying, the samples were prepared for application to the MALDI target by one of two methods. EITHER Reconstitution Solvent A

consisting of 700/270/30 (V/V/V) acetonitrile/water/10% trifluoroacetic acid, was added to the sample, and only a portion of the sample was then mixed 1/1 (V/V) with the CHCA matrix solution, OR the entire sample was reconstituted in Solution B which was a 1/1 (V/V) mixture of Reconstitution Solvent A and CHCA matrix solution. The method used for each sample has been provided in the summary spreadsheets attached to this report. Portions (0.5-0.7 μL) of the sample/matrix mixtures were applied to two different positions on the MALDI target. MALDI TOF/TOF MS (AB Sciex, formerly Applied Biosystems, Inc., 4700 mass spectrometer) survey spectra were obtained from one of the positions for each sample in order to determine the laser setting to be used in the automated spectrum acquisition from a second un-interrogated application of the sample. The 10 most intense ions were analyzed by MS/MS (order of analysis was most intense to least intense).

Spot Set MS Analysis Start Date	Batch Number	Samples
5/19/2016	1	Digests of well-stained PUPA Samples
5/24/2016	2	Digests of well-stained LARVA and MALE Samples
5/26/2016	3	Digests of well-stained EGG Samples
6/10/2016	4	Digests of well-stained FEMALE Samples
6/14/2016	5	Digests of remaining poorly-stained Samples

In the case of samples for which significant ions in the MS spectrum were not automatically selected for MS/MS ions (although they should have been based on ion intensity), additional MS/MS were obtained from the second application of the sample using manual selection of the precursor ions of interest for MS/MS. (Analyses of **6** samples were repeated for this reason.)

In the case of many of the protein digests from "Egg" samples, there was an abundance of higher molecular weight peptides which were not analyzed in the first automated analysis because the masses of the peptides were greater than the 2500-Da mass cut-off for inclusion in the precursor ion list for automated MS/MS. Consequently, all of the samples in Batch 3 from "Egg" were analyzed a second time with an automated method which specified selection of precursor ions from 2200-4500 Da. (**37 digests from "Egg" samples were analyzed a second time**.)

The spectra were evaluated by the Analyst for spectrum quality and mass accuracy. Most of the samples in this set contained only very low levels of trypsin autolysis peptides. Consequently, all of the samples were analyzed with the default external calibration which was based on analysis of peptide calibration standards applied to a minimum of 6 positions which surrounded the samples on the target. The MS spectra mass error was assessed on the basis of the differences between the precursor ion mass in the original MS spectrum *vs*. the mass observed for the residual amount of precursor ion seen in the corresponding MS/MS spectrum. Generally, the precursor ion masses found in the MS and MS/MS spectra were within less than 200 ppm of each other.

For these samples, spectra were processed and batch analyzed in the "Combined MS plus MS/MS" mode with the Applied Biosystems GPS Explorer software (vers. 3.6). Database searches were performed with Matrix Science's Mascot search engine v. 2.4 (www.matrixscience.com) on an in-house server (the "Old Mascot Server") against the NCBInr "Blat 20160224" database and the NCBInr Insects database which had been added to the server February 20, 2015. The database search parameters are shown in the following table. In some cases, scores for an assignment were found to be improved if deamidation at Aspargines or Acetylation of the protein N-terminus was added to the variable modifications permitted for the search. These were noted on the Mascot Reports for the *B. lat* searches which have been printed and will be sent to Dr. Chang.

Final Database Search Parameters **

Enzyme	# Missed Cleavages	Mass Error Tolerances		Mascot Scores Required for $p < 0.05$			
		Precursor Ions	MS/MS Fragment Ions	NCBInr "Blat 20160224"		NCBInr Insects (02202015)	
				Total Protein Score	Individual MS/MS Ion Score	Total Protein Score	Individual MS/MS Ion Score
Trypsin	≤ 2	± 200 ppm	± 0.2 Da	> 58	> ~32	>76	>49

**Carbamidomethylation of Cysteines was a required modification. The permitted variable modifications were Methionine oxidation and Pyroglutamate formation at peptide N-terminal Glutamic acids and peptide N-terminal Glutamines.

The GPS Explorer reports generated from the Matrix Science Mascot reports were saved as PDF files. These PDF files will be sent to Dr. Chang as email attachments. Excel Spreadsheets for the GPS Explorer Protein reports will also be sent to Dr. Chang as email attachments at the same time. These spreadsheets, which contain the names and database accession numbers for the tentatively matched proteins, will provide an easy route to preparing the final summary of the data for all of the samples.

Printed copies of the Mascot Reports for the results of the searches against the *B. lat.* database will be provided to Dr. C. Goodman for transfer to Dr. S. Chang. The Protein Views for the major protein matches have been included in the Mascot Reports. The percent sequence recoveries and the full protein sequences appear in the Protein Views. Only a few selected Mascot Reports from the searches *vs.* the 2015 NCBInr Insect database have been printed. These were for samples for which the MS and MS/MS spectra were of high quality, the *B. lat.* database provided only very weak assignments, and better matches were made with other insects (in particular *B. dor.*). Additional printed Mascot reports for the searches *vs.* the Insects database can be provided upon request.

A flash drive will be provided containing the exported ".t2d" files for the spectra. (The ".t2d" files require the "Data Explorer" program for viewing.) The peak lists from the spectra have also been exported in Mascot Generic Format (mgf) and saved to the flash drive as well if Dr. Chang wishes to have them. Copies of the GPS Explorer report pdfs have been included on the flash drive. Excel Spreadsheets for the Protein Summaries generated by the GPS Explorer software have been created (but not heavily edited into pretty form) and saved to the flash drive to aid the Investigator in preparing her summary tables. Finally, the original spectra for these samples as well as those in the set submitted in February of 2016, have been archived on the flash drive. The original data can only be viewed on the 4700 MALDI TOF/TOF mass spectrometer, so these files should not be opened by the Investigators.

All data has also been archived in the Proteomics Center. All Matrix Science Mascot reports are stored on the Proteomics Center server.

Copies of the printed spectra can be supplied upon request

Sample Processing and Analytical Methods:

All aqueous solutions were prepared with 18 Mohm x cm water produced by a Millipore Milli-Q Synthesis A10 water purification system. Sigma-Aldrich was the source for alpha-cyano-4-hydroxycinnamic acid (CHCA, Fluka 70990, mass-spec grade) and the peptide components of the Mass Spectrometer calibration mix (see below). Mass Spec-grade acetonitrile (Optima, A955-4) was from Fisher Scientific. Sequencing-grade trifluoroacetic acid (TFA) was from Burdick and Jackson (BB360P050). The sources of other reagents or materials have been noted in the text

The trypsin digests of Coomassie Blue-stained 2-D gel plugs were lyophilized dry. The dried digests were reconstituted in 10 µL water and dried a second time to further reduce the amount of contaminating volatile buffer salts. On the day following the overnight drying, the samples were prepared for application to the MALDI target by one of two methods. EITHER Reconstitution Solvent A consisting of 700/270/30 (V/V/V)

acetonitrile/water/10% trifluoroacetic acid, was added to the sample, and only a portion of the sample was then mixed 1/1 (V/V) with the CHCA matrix solution, OR the entire sample was reconstituted in Solution B which was a 1/1 (V/V) mixture of Reconstitution Solvent A and CHCA matrix solution. The method used for each sample has been provided in the summary spreadsheets attached to this report. Portions (0.5-0.7 µL) of the sample/matrix mixtures were applied to two different positions on the MALDI target (polished stainless steel target, ABI01-192-6-AB). Crystallization of the mixtures on the target proceeded under ambient conditions. The crystals were not washed. CHCA matrix solution was prepared at a concentration of 10 mg/mL in 600/370/30 (V/V/V) acetonitrile/water/10% TFA.

Spectra for the digests were acquired on an Applied Biosystems Inc. (now AB Sciex) 4700 MALDI TOF/TOF mass spectrometer with a 355 nm Nd:YAG laser (200-Hz) in the positive ion delayed-extraction reflector MS or positive ion MS/MS mode. (Instrument maintenance and installation of new laser and detector: 4/18-4/22/2016. Additional instrument maintenance and final tuning: 6/1-6/3/2016.) The MS spectra were acquired over the mass range 835-4500 Da. MS spectra were the result of 100 20-shot sub-spectra (first 2 shots of each sub-spectrum rejected). Laser settings have been noted in the Table below. An accelerating voltage of 20 kV combined with a 70% extraction grid voltage and an extraction delay time of 550 nsec (focus mass at 2000 Da), resulted in mass resolving powers of ~8000 at 900 Da, ~17000 at 2000 Da, and ~13000 at 3650 Da. The detector voltage multiplier (DVM) was varied as indicated for the different batches of samples. The digitizer settings included a bin size of 0.5 ns, a full-scale of 200 mV, and an input bandwidth of 500 MHz. The Global External (Default) Calibration and plate modeling were based on internally calibrated spectra for the Mass Spectrometer calibration solution (see below) spotted on at least 6 positions surrounding the sample spots on the target. Spectra were processed with Applied Biosystems' 4000 Series Explorer software (version 3.6).

Spot Set Analysis Start Date	Batch Number	Samples	MS Settings			MS/MS Settings		
			Laser	DVM	mV full-scale	Laser	DVM	mV full-scale
5/19/2016	1	Digests of well-stained PUPA Samples	1700	0.9	200	2700 to 2900	0.97	100
5/24/2016	2	Digests of well-stained LARVA and MALE Samples	2100	0.9	200	3500	0.97	100
5/26/2016	3	Digests of well-stained EGG Samples	2100	0.9	200	3500	0.97	100
6/10/2016	4	Digests of well-stained FEMALE Samples	2200	0.95	200	3500	1.0	200
6/14/2016	5	Digests of remaining poorly-stained Samples	2200	0.95	200	3500	1.0	200

The Mass Spectrometer calibration solution was prepared from stock solutions (prepared 6/19/2015) of peptides obtained from Sigma-Aldrich. The peptide concentrations shown in the table below were for the solutions actually applied to the target, that is, after mixing with CHCA matrix. The volume of the applied solution was 0.5-0.7 µL. Comparison of the signal intensities for the sample peptides with those for the known amounts of the standards shown below may help in estimating the amounts of the sample peptides.

Peptide	$[M+H]^+$ Monoisotopic (Da)	fmole Peptide/µL in Spotting Solution
des-Arg1 Bradykinin	904.468	100
Angiotensin I	1296.685	200
[Glu1] Fibrinopeptide B	1570.677	130
ACTH [1-17]	2093.087	200
ACTH [18-39]	2465.199	150
Neurotensin	1672.9175	5
ACTH [7-38]	3657.929	**1000**

The 1-KV peptide unimolecular decomposition spectra, or **MS/MS, were obtained <u>without</u> any CID gas** and with metastable ion suppression. Resolution for ions in the MS/MS spectra was about 5000 at 1300 Da. The mass window for precursor ion selection was from 2.0 Da below the precursor ion mass to 3.0 Da above the precursor ion mass. MS/MS spectra were the result of <u>at least</u> 3500 (175 20-shot sub-spectra, no shots rejected). External mass calibration for the MS/MS was based on fragment ion masses from the peptide ACTH [18-39] ($[M+H]^+$ 2465.199 Da) in the Mass Spectrometer calibration solution spotted on at least 6 positions surrounding the sample spots on the target. MS/MS analyses for sample peptides were acquired for the 10 most intense ions (most intense first, minimum S/N 15 counts). Commonly observed contaminant ions and trypsin autolysis peptides in the sample digests were excluded from MS/MS analysis. Ions excluded from MS/MS analysis in the automated mode are listed in the table below.

Spot Set Analysis Start Date	Batch Number	Samples	MS Ions Excluded from MS/MS in Automated Acquisition Method (Interpretation Method) Minimum S/N Filter was 15 for all analyses.
5/19/2016	1	Digests of well-stained PUPA Samples	568.14, 795-895, 1045.564, 1794.8, 1826.8, 1940.935, 2211.105, 2225.12, 2283.181, 2284.19, 2299.18, 2300.18, 2807.315, 3337.758, 2501-4501<br>Na and K adducts or Oxidized forms (± 0.2 Da)
5/24/2016	2	Digests of well-stained LARVA and MALE Samples	568.14, 795-895, 1045.564, 1794.8, 1826.8, 1940.935, 2211.105, 2225.12, 2283.181, 2284.19, 2299.18, 2300.18, 2807.315, 3337.758, 2501-4501<br>Na and K adducts or Oxidized forms (± 0.2 Da)
5/26/2016	3	Digests of well-stained EGG Samples	<u>1st Analysis:</u><br>568.14, 795-895, 1045.564, 1794.8, 1826.8, 2211.105, 2225.12, 2283.181, 2284.19, 2299.18, 2300.18, 2807.315, 3337.758, 2501-4501 (the 1940.935 trypsin autolysis peptide mass was deleted from the exclusion list)<br>Na and K adducts or Oxidized forms (± 0.2 Da)<br><u>2nd Analysis:</u><br>Ions < 2200 Da and >4500 Da<br>Na and K adducts or Oxidized forms (± 0.2 Da)
6/10/2016	4	Digests of well-stained FEMALE Samples	568.14, 1045.564, 1794.8, 1826.8, 1940.935, 2211.105, 2225.12, 2283.181, 2284.19, 2299.18, 2300.18, 2807.315, 3337.758, 2800-4500<br>Na and K adducts or Oxidized forms (± 0.2 Da)
6/14/2016	5	Digests of remaining poorly-stained Samples	568.14, 1045.564, 1794.8, 1826.8, 2211.105, 2225.12, 2283.181, 2284.19, 2299.18, 2300.18, 2807.315, 3337.758, 2800-4500<br>Na and K adducts or Oxidized forms (± 0.2 Da)

<u>Spectra were processed and batch analyzed in the "combined MS and MS/MS" mode with Applied Biosystems' GPS Explorer software (vers. 3.6).</u> Database searches were performed with Matrix Science's Mascot search engine v. 2.4 (<u>www.matrixscience.com</u>) residing on the OLD in-house Mascot server. Searches were performed against the *Bactrocera latifrons* database downloaded from the NCBInr database on February 24, 2016. The final search criteria were as indicated in the Work Summary section above. The source protein molecular weight was not limited in any of the searches. The mass error tolerance for precursor ions was ± 200 ppm. The mass error tolerance was ± 0.2 Da for MS/MS Fragment Ions. The commonly observed trypsin autolysis peptide masses were excluded (with a mass tolerance of 100 ppm) from the MS spectra peak lists (832.3, 842.51, 870.54, 1045.5642, 1826.6, 2211.1046, 2239.1359, 2283.1807, 2299.1756, 2807.31, 3337.758 Da). The signal/noise

(S/N) threshold was set to 20 for creating the peak lists from the MS spectra and to 10 for preparing the MS/MS spectra peak lists. A maximum of 500 ions could be selected from an MS spectrum for searching while the MS/MS peak lists were limited to 250 ions.

Shown below are the parameters utilized with Applied Biosystems' "Launch Peaks to Mascot" program to generate the MS and MS/MS peak lists in the "mgf" format. In this format the data also may be submitted by the Investigators directly to the Mascot "MS/MS Ion Search" program at the public website (www.matrixscience.com). Note that no ions were excluded from the mgf formatted MS data peak lists. The ".mgf" files have been saved in a folder on a flash drive for Dr. Chang.

Parameters for exporting the spectrum data in the Mascot Generic Format (".mgf" files)	
MS Data	MS/MS Data
Input MS mass range: starting mass-4500 Da	Input mass range: from 60 Da to 15 Da below mass of the precursor ion
No Ion Exclusion List	No Ion Exclusion List
Peak Density: max 50 ions/200 Da	Peak Density: max 50 ions/200 Da
Minimum S/N: 20	Minimum S/N: 10
Minimum Area: 1000	Minimum Area: 500
Maximum Number of Ions: 500	Maximum Number of Ions: 250

Analyst's Signature/Date: /July 8, 2016

Beverly DaGue

D. Stanley, C. Goodman, and Stella Chang, Received 05/12/16
EGG SAMPLES FOR MASS SPEC ANALYSIS

The dried digest samples were prepared for application to the MALDI target by one of two methods.
EITHER Reconstitution Solvent A consisting of 700/270/30 (V/V/V) acetonitrile/water/10% trifluoroacetic acid, was added to the sample, and only a portion of the sample was then mixed 1/1 (V/V) with the CHCA matrix solution,
OR the entire sample was reconstituted in Solution B which was a 1/1 (V/V) mixture of Reconstitution Solvent A and CHCA matrix solution.

Count	Spot ID	Images	Score	Reconstitution Volume (µL)	Reconstitution Solvent	µL Sample in Solvent A mixed with equal Volume of CHCA	Analysis Batch #	Target Position 1ST ANALYSIS	Database Search MALDI Well I.D. 1ST ANALYSIS	Blat Search URL 1ST ANALYSIS	NCBInr Insects Search URL 1ST ANALYSIS	Target Position 2ND ANALYSIS	Database Search MALDI Well I.D. 2ND ANALYSIS	Blat Search URL 2ND ANALYSIS	NCBInr Insects Search URL 2ND ANALYSIS
1	LAT E-ID104	1	4	8	A	1.5	3	B3	125487	F20160528 FF020797		E3	125559	F20160531 FF020832	
2	LAT E-ID105	1	4	8	A	1.5	3	B5	125489	F20160528 FF020799		E5	125561	F20160528 FF020831	
3	LAT E-ID112	1	2	2	B		5	E3	126359	F20160614 FF021027	F20160615 FF021087				
4	LAT E-ID113	1	4	2	A	1	3	B7	125491	F20160528 FF020801		E7	125563	F20160531 FF020835	
5	LAT E-ID116	1	4	2	A	1.5	3	B9	125493	F20160528 FF020803	F20160615 FF021138	E9	125565	F20160531 FF020837	F20160615 FF021137
6	LAT E-ID121	1	4	4	A	1.5	3	B11	125495	F20160528 FF020805		E11	125567	F20160531 FF020839	
7	LAT E-ID128	1	4	4	A	1.5	3	B13	125497	F20160528 FF020807		E13	125569	F20160531 FF020841	
8	LAT E-ID129	1	4	2	A	1	3	B15	125499	F20160528 FF020809		E15	125571	F20160531 FF020843	
9	LAT E-ID157	1	1	2	B		5	E4	126360	F20160614 FF021028	F20160615 FF021089				
10	LAT E-ID183	1	1	2	B		5	E5	126361	F20160614 FF021029	F20160615 FF021090				
11	LAT E-ID252	1	4	3	A	1.5	3	B17	125501	F20160528 FF020811		E17	125573	F20160531 FF020845	
12	LAT E-ID257	1	4	4	A	1.5	3	B19	125503	F20160528 FF020813		E19	125575	F20160531 FF020847	
13	LAT E-ID267	1	4	8	A	1.5	3	B21	125505	F20160528 FF020815		E21	125577	F20160531 FF020849	
14	LAT E-ID268	1	4	8	A	1.5	3	B2	125486	F20160528 FF020796		E2	125558	F20160531 FF020867	
15	LAT E-ID276	1	4	8	A	1.5	3	B4	125488	F20160528 FF020798		E4	125560	F20160531 FF020833	
15	LAT E-ID276											F10	125590	F20160531 FF020859	
16	LAT E-ID277	1	4	4	A	1.5	3	B6	125490	F20160528 FF020800		E6	125562	F20160531 FF020834	
16	LAT E-ID277											F12	125592	F20160531 FF020861	
17	LAT E-ID281	1	4	4	A	1.5	3	B8	125492	F20160528 FF020802		E8	125564	F20160531 FF020836	
18	LAT E-ID282	1	4	6	A	1.5	3	B10	125494	F20160528 FF020804	F20160615 FF021140	E10	125566	F20160531 FF020838	F20160615 FF021139
19	LAT E-ID291	1	4	4	A	1.5	3	B12	125496	F20160528 FF020806		E12	125568	F20160531 FF020840	
20	LAT E-ID293	1	4	4	A	1.5	3	B14	125498	F20160528 FF020808		E14	125570	F20160531 FF020842	

D. Stanley, C. Goodman, and Stella Chang, Received 05/12/16
EGG SAMPLES FOR MASS SPEC ANALYSIS

The dried digest samples were prepared for application to the MALDI target by one of two methods.
EITHER Reconstitution Solvent A consisting of 700/270/30 (V/V/V) acetonitrile/water/10% trifluoroacetic acid, was added to the sample, and only a portion of the sample was then mixed 1/1 (V/V) with the CHCA matrix solution,
OR the entire sample was reconstituted in Solution B which was a 1/1 (V/V) mixture of Reconstitution Solvent A and CHCA matrix solution.

Count	Spot ID	Images	Score	Reconstitution Volume (µL)	Reconstitution Solvent	µL Sample in Solvent A mixed with equal Volume of CHCA	Analysis Batch #	Target Position 1ST ANALYSIS	Database Search MALDI Well I.D. 1ST ANALYSIS	Blat Search URL 1ST ANALYSIS	NCBInr Insects Search URL 1ST ANALYSIS	Target Position 2ND ANALYSIS	Database Search MALDI Well I.D. 2ND ANALYSIS	Blat Search URL 2ND ANALYSIS	NCBInr Insects Search URL 2ND ANALYSIS
21	LAT E-ID307	1	4	6	A	1.5	3	B16	125500	F20160528 FF020810		E16	125572	F20160531 FF020844	
22	LAT E-ID308	1	4	2	A	1.5	3	B18	125502	F20160528 FF020812		E18	125574	F20160531 FF020846	
23	LAT E-ID312	1	4	4	A	1.5	3	B20	125504	F20160528 FF020814		E20	125576	F20160531 FF020848	
24	LAT E-ID313	1	4	4	A	1.5	3	B22	125506	F20160528 FF020816		E22	125578	F20160531 FF020850	
25	LAT E-ID316	1	4	8	A	1.5	3	C3	125511	F20160528 FF020818		F3	125583	F20160531 FF020852	
26	LAT E-ID317	1	4	4	A	1.5	3	C5	125513	F20160528 FF020820		F5	125585	F20160531 FF020854	
27	LAT E-ID322	1	4	3	A	1.5	3	C7	125515	F20160528 FF020822		F7	125587	F20160531 FF020856	
28	LAT E-ID326	1	4	6	A	1.5	3	C9	125517	F20160528 FF020824		F9	125589	F20160531 FF020858	
29	LAT E-ID327	1	4	4	A	1.5	3	C11	125519	F20160528 FF020825		F11	125591	F20160531 FF020860	
30	LAT E-ID366	1	4	3	A	1.5	3	C13	125521	F20160528 FF020826		F13	125593	F20160531 FF020862	
31	LAT E-ID564	1	4	8	A	1.5	3	C15	125523	F20160528 FF020827		F17	125597	F20160531 FF020863	
32	LAT E-ID628	1	4	3	A	1.5	3	C17	125525	F20160528 FF020828		F19	125599	F20160531 FF020864	
33	LAT E-ID633	1	4	12	A	1.5	3	C19	125527	F20160528 FF020829		F21	125601	F20160531 FF020865	
34	LAT E-ID640	1	4	3	A	1.5	3	C21	125529	F20160528 FF020830		F23	125603	F20160531 FF020866	
35	LAT E-ID643	1	3	2	A	1	5	E6	126362	F20160614 FF021030 See also: F20160616 FF021198	F20160615 FF021091				
36	LAT E-ID646	1	4	8	A	1.5	3	C2	125510	F20160528 FF020817		F2	125582	F20160531 FF020851	
37	LAT E-ID650	1	4	10	A	1.5	3	C4	125512	F20160528 FF020819	F20160615 FF021170	F4	125584	F20160531 FF020853	F20160615 FF021169
38	LAT E-ID685	1	3	3	A	1.5	3	C6	125514	F20160528 FF020821		F6	125586	F20160531 FF020855	
39	LAT E-ID98	1	4	4	A	1.5	3	C8	125516	F20160528 FF020823		F8	125588	F20160531 FF020857	

D. Stanley, C. Goodman, and Stella Chang, Received 05/12/16

LARVA SAMPLES FOR MASS SPEC ANALYSIS

The dried digest samples were prepared for application to the MALDI target by one of two methods.
EITHER Reconstitution Solvent A consisting of 700/270/30 (V/V/V) acetonitrile/water/10% trifluoroacetic acid, was added to the sample, and only a portion of the sample was then mixed 1/1 (V/V) with the CHCA matrix solution,
OR the entire sample was reconstituted in Solution B which was a 1/1 (V/V) mixture of Reconstitution Solvent A and CHCA matrix solution.

Count	Spot ID	Images	Score	Reconstitution Volume (µL)	Reconstitution Solvent	µL Sample in Solvent A mixed with equal Volume of CHCA	Analysis Batch #	Target Position 1ST ANALYSIS	Database Search MALDI Well I.D. 1ST ANALYSIS	Blat Search URL 1ST ANALYSIS	NCBInr Insects Search URL 1ST ANALYSIS	Target Position 2ND ANALYSIS	Database Search MALDI Well I.D. 2ND ANALYSIS	Blat Search URL 2ND ANALYSIS	NCBInr Insects Search URL 2ND ANALYSIS
1	LAT L-130	2	4	3	A	1.5	2	E2	125360	F20160528 FF020767					
2	LAT L-136	2	2	2	B		5	E7	126363	F20160614 FF021031	F20160615 FF021092				
3	LAT L-372	2	4	3	A	1.5	2	E4	125362	F20160528 FF020768					
4	LAT L-379	2	4	3	A	1.5	2	E6	125364	F20160528 FF020769					
5	LAT L-395	2	4	4	A	1.5	2	E8	125366	F20160528 FF020770					
6	LAT L-401	2	1	2	B		5	E8	126364	F20160614 FF021032	F20160615 FF021093				
7	LAT L-407	2	2	2	A	1	2	E10	125368	F20160528 FF020771					
8	LAT L-416	2	1	2	B		5	E9	126365	F20160614 FF021033	F20160615 FF021094				
9	LAT L-419	2	4	4	A	1.5	2	E12	125370	F20160528 FF020772					
10	LAT L-426	2	1	2	B		5	E10	126366	F20160614 FF021034	F20160615 FF021096				
11	LAT L-454	2	2	2	B		2	E14	125372	F20160528 FF020773		B14	125300	F20160528 FF020765	
12	LAT L-485	2	2	2	B		2	E16	125374	F20160528 FF020774					
13	LAT L-551	2	4	2	A	1	2	E18	125376	F20160528 FF020775					
14	LAT L-566	2	1	2	B		2	E20	125378	F20160528 FF020776					
15	LAT L-571	2	1	2	B		2	E22	125380	F20160528 FF020777					
16	LAT L-59	2	4	3	A	1.5	2	F3	125385	F20160528 FF020778					
17	LAT L-674	2	1	2	B		5	E11	126367	F20160614 FF021035	F20160615 FF021099				
18	LAT L-679	2	2	2	A	1	2	F5	125387	F20160528 FF020779					
19	LAT L-708	2	4	3	A	1.5	2	F7	125389	F20160528 FF020780					
20	LAT L-710	2	3	2	A	1	2	F9	125391	F20160528 FF020781					
21	LAT L-721	2	1	2	B		5	E12	126368	F20160614 FF021036	F20160615 FF021101				
22	LAT L-739	2	4	3	A	1.5	2	F11	125393	F20160528 FF020782					
23	LAT L-2001	3	4	2	A	1	2	F13	125395	F20160528 FF020783					

D. Stanley, C. Goodman, and Stella Chang, Received 05/12/16
LARVA SAMPLES FOR MASS SPEC ANALYSIS

The dried digest samples were prepared for application to the MALDI target by one of two methods.
EITHER Reconstitution Solvent A consisting of 700/270/30 (V/V/V) acetonitrile/water/10% trifluoroacetic acid, was added to the sample, and only a portion of the sample was then mixed 1/1 (V/V) with the CHCA matrix solution,
OR the entire sample was reconstituted in Solution B which was a 1/1 (V/V) mixture of Reconstitution Solvent A and CHCA matrix solution.

Count	Spot ID	Images	Score	Reconstitution Volume (µL)	Reconstitution Solvent	µL Sample in Solvent A mixed with equal Volume of CHCA	Analysis Batch #	Target Position 1ST ANALYSIS	Database Search MALDI Well I.D. 1ST ANALYSIS	Blat Search URL 1ST ANALYSIS	NCBInr Insects Search URL 1ST ANALYSIS	Target Position 2ND ANALYSIS	Database Search MALDI Well I.D. 2ND ANALYSIS	Blat Search URL 2ND ANALYSIS	NCBInr Insects Search URL 2ND ANALYSIS
24	LAT L-2120	3	4	3	A	1.5	2	F15	125397	F20160528 FF020784					
25	LAT L-2187	3	4	6	A	1.5	2	F17	125399	F20160528 FF020785					
26	LAT L-2367	3	1	2	B		5	E13	126369	F20160614 FF021037	F20160615 FF021102				
27	LAT L-2483	3	3	4	A	1.5	2	F19	125401	F20160528 FF020786	F20160626 FF021324				
28	LAT L-2487	3	4	2	A	1	2	F21	125403	F20160528 FF020787	F20160626 FF021325				
29	LAT L-2496	3	4	3	A	1.5	2	F23	125405	F20160528 FF020788					
30	LAT L-2569	3	4	3	A	1.5	2	G3	125409	F20160528 FF020789		D3	125337	F20160528 FF020766	
31	LAT L-2590	3	1	2	B		5	E14	126370	F20160614 FF021038	F20160615 FF021104				
32	LAT L-2634	3	4	4	A	1.5	2	G5	125411	F20160528 FF020790					
33	LAT L-2652	3	2	2	B		5	E15	126371	F20160614 FF021039	F20160615 FF021107				

D. Stanley, C. Goodman, and Stella Chang, Received 05/12/16

PUPA SAMPLES FOR MASS SPEC ANALYSIS

The dried digest samples were prepared for application to the MALDI target by one of two methods.
EITHER Reconstitution Solvent A consisting of 700/270/30 (V/V/V) acetonitrile/water/10% trifluoroacetic acid, was added to the sample, and only a portion of the sample was then mixed 1/1 (V/V) with the CHCA matrix solution,
OR the entire sample was reconstituted in Solution B which was a 1/1 (V/V) mixture of Reconstitution Solvent A and CHCA matrix solution.

Count	Spot ID	Images	Score	Reconstitution Volume (µL)	Reconstitution Solvent	µL Sample in Solvent A mixed with equal Volume of CHCA	Analysis Batch #	Target Position 1ST ANALYSIS	Database Search MALDI Well I.D. 1ST ANALYSIS	Blat Search URL 1ST ANALYSIS	NCBInr Insects Search URL 1ST ANALYSIS	Target Position 2ND ANALYSIS	Database Search MALDI Well I.D. 2ND ANALYSIS	Blat Search URL 2ND ANALYSIS	NCBInr Insects Search URL 2ND ANALYSIS
1	LAT P-ID2070	4	4	8	A	2	1	B4	124894	F20160520 FF020658	F20160622 FF021284				
2	LAT P-ID2074	4	1	2	B		5	E16	126372	F20160614 FF021040	F20160615 FF021108				
3	LAT P-ID2262	4	4	4	A	2	1	B6	124896	F20160520 FF020659					
4	LAT P-ID2304	4	2	2	A	1	1	B8	124898	F20160520 FF020660	F20160622 FF021286				
5	LAT P-ID2305	4	4	4	A	2	1	B12	124902	F20160520 FF020662					
6	LAT P-ID2322	4	4	4	A	2	1	B10	124900	F20160520 FF020661					
7	LAT P-ID2435	4	4	4	A	2	1	B16	124906	F20160520 FF020664					
8	LAT P-ID2436	4	4	4	A	2	1	B14	124904	F20160520 FF020663					
9	LAT P-ID2466	4	3	2	B		5	E17	126373	F20160614 FF021041	F20160615 FF021110				
10	LAT P-ID2558	4	1	2	B		5	E18	126374	F20160614 FF021042	F20160615 FF021112				
11	LAT P-ID211	5	1	2	B		5	E19	126375	F20160614 FF021043	F20160615 FF021115				
12	LAT P-ID500	5	4	4	A	2	1	B18	124908	F20160520 FF020666	F20160622 FF021292	B17	124907	F20160520 FF020665	F20160622 FF021291
13	LAT P-ID519	5	4	2	A	1	1	B24	124914	F20160520 FF020670	F20160622 FF021296	B23	124913	F20160520 FF020671	
14	LAT P-ID1511	5	4	8	A	2	1	B20	124910	F20160520 FF020667	F20160622 FF021293				
15	LAT P-ID1654	5	4	2	A	1	1	B22	124912	F20160520 FF020669	F20160622 FF021295	B21	124911	F20160520 FF020668	F20160622 FF021294

D. Stanley, C. Goodman, and Stella Chang, Received 05/12/16
MALE SAMPLES FOR MASS SPEC ANALYSIS

The dried digest samples were prepared for application to the MALDI target by one of two methods.
EITHER Reconstitution Solvent A consisting of 700/270/30 (V/V/V) acetonitrile/water/10% trifluoroacetic acid, was added to the sample, and only a portion of the sample was then mixed 1/1 (V/V) with the CHCA matrix solution,
OR the entire sample was reconstituted in Solution B which was a 1/1 (V/V) mixture of Reconstitution Solvent A and CHCA matrix solution.

Count	Spot ID	Images	Score	Reconstitution Volume (µL)	Reconstitution Solvent	µL Sample in Solvent A mixed with equal Volume of CHCA	Analysis Batch #	Target Position 1ST ANALYSIS	Database Search MALDI Well I.D. 1ST ANALYSIS	Blat Search URL 1ST ANALYSIS	NCBInr Insects Search URL 1ST ANALYSIS	Target Position 2ND ANALYSIS	Database Search MALDI Well I.D. 2ND ANALYSIS	Blat Search URL 2ND ANALYSIS	NCBInr Insects Search URL 2ND ANALYSIS
1	LAT M-ID1251	6	4	2	A	1	2	G7	125413	F20160528 FF020791					
2	LAT M-ID1261	6	4	4	A	1.5	2	G9	125415	F20160528 FF020792					
3	LAT M-ID1288	6	1	2	B		5	E20	126376	F20160614 FF021044	F20160615 FF021117				
4	LAT M-ID1344	6	1	2	B		5	E21	126377	F20160614 FF021045	F20160615 FF021119				
5	LAT M-ID1535	6	4	4	A	1.5	2	G11	125417	F20160528 FF020793	F20160626 FF021331				
6	LAT M-ID1617	6	1	2	B		5	E22	126378	F20160614 FF021046	F20160615 FF021121				
7	LAT M-ID1475	7	4	4	A	1.5	2	G13	125419	F20160528 FF020794	F20160626 FF021332				
8	LAT M-ID1561	7	4	4	A	1.5	2	G15	125421	F20160528 FF020795	F20160626 FF021333				

D. Stanley, C. Goodman, and Stella Chang, Received 05/12/16
FEMALE SAMPLES FOR MASS SPEC ANALYSIS

The dried digest samples were prepared for application to the MALDI target by one of two methods.
EITHER Reconstitution Solvent A consisting of 700/270/30 (V/V/V) acetonitrile/water/10% trifluoroacetic acid, was added to the sample, and only a portion of the sample was then mixed 1/1 (V/V) with the CHCA matrix solution,
OR the entire sample was reconstituted in Solution B which was a 1/1 (V/V) mixture of Reconstitution Solvent A and CHCA matrix solution.

Count	Spot ID	Images	Score	Reconstitution Volume (µL)	Reconstitution Solvent	µL Sample in Solvent A mixed with equal Volume of CHCA	Analysis Batch #	Target Position 1ST ANALYSIS	Database Search MALDI Well I.D. 1ST ANALYSIS	Blat Search URL 1ST ANALYSIS	NCBInr Insects Search URL 1ST ANALYSIS	Target Position 2ND ANALYSIS	Database Search MALDI Well I.D. 2ND ANALYSIS	Blat Search URL 2ND ANALYSIS	NCBInr Insects Search URL 2ND ANALYSIS
1	LAT F-ID223	8	4	4	A	1.5	4	E5	126163	F20160610 FF020986					
2	LAT F-ID226	8	3	2	A	1	4	E6	126164	F20160610 FF020987					
3	LAT F-ID299	8	4	4	A	1.5	4	E7	126165	F20160610 FF020988					
4	LAT F-ID363	8	1	2	B		4	E8	126166	F20160610 FF020989					
5	LAT F-ID403	8	4	4	A	1.5	4	E9	126167	F20160610 FF020990					
6	LAT F-ID533	8	4	4	A	1.5	4	E10	126168	F20160610 FF020991	F20160615 FF021105				
7	LAT F-ID541	8	4	4	A	1.5	4	E11	126169	F20160610 FF020992					
8	LAT F-ID548	8	3	3	A	1.5	4	E12	126170	F20160610 FF020993					
9	LAT F-ID625	8	1	2	B		4	E13	126171	F20160610 FF020994	F20160615 FF021111				
10	LAT F-ID654	8	1	2	B		4	E14	126172	F20160610 FF020995					
11	LAT F-ID779	8	3	3	A	1.5	4	E15	126173	F20160610 FF020996		B15	126101	F20160610 FF021020	
12	LAT F-ID1051	9	1	2	B		4	E16	126174	F20160610 FF020997					
13	LAT F-ID1253	9	4	2	A	1	4	E17	126175	F20160610 FF020998					
14	LAT F-ID903	9	1	2	B		4	E18	126176	F20160610 FF020999					
15	LAT F-ID911	9	4	2	A	1	4	E19	126177	F20160610 FF021000					
16	LAT F-ID972	9	4	3	A	1.5	4	E20	126178	F20160610 FF021001	F20160615 FF021124				
17	LAT F-ID978	9	3	2	A	1	4	E21	126179	F20160610 FF021002	F20160615 FF021125				

Charles W. Gehrke Proteomics Center<br>Research Core Facilities<br>University of Missouri-Columbia	212 Life Sciences Center<br>1201 East Rollins Road<br>Columbia, MO 65211

REQUEST FOR MASS SPECTRAL ANALYSIS

Name:	Cindy Goodman/Stella Chang	Phone:	573-876-8303
Email:	goodmanc@missouri.edu	Date:	5/12/2016
Campus address:	1503 S. Providence Rd	PI or Authorized Signature:	D Stanley
PI:	David Stanley		
MoCode:	DN778 Dept: USDA, ARS, BCIRL		

**Sample information**	
Sample type:	Trypsin digests of 2D Gel spots
Source:	Solanum fruit fly (Bactrocera latifrons) - eggs, larvae, pupae, adults
Further Information:	
PTM:	
Amount:	
Purity:	
Mol Wt/pI	
Detection method:	
Prior treatment:	Trypsin digest (as previously described)
Other modifications?	
Toxicity:	
Stability:	

Objectives of analyses or other comments:

To determine protein identities..

**Sample designation:**	
1)	See sample list
2)	
3)	
4)	
5)	
6)	

TYPE OF ANALYSIS REQUESTED	Number	Comments
Manual or robotic gel excision (hours)		
In-gel digestion - 1D gel band(s)		
In-gel digestion - 2D gel spot(s)		
In-gel digestion - 96 well plates (2D spots)		
Solution digest		
ZIP tip peptide clean-up and concentration		
MALDI-TOF MS		
Automated MALDI-TOF MS		
4700 MALDI TOF-TOF MS & MS/MS	112	
Automated 4700 MALDI TOF-TOF		
Nanospray(ionspray) QqTOF MS		
Nanospray(ionspray) QqTOF MS/MS		
Data analysis (*de novo* sequencing etc.)		
LTQ Orbitrap or 6520 QTOF LC-MS		
LTQ Orbitrap or 6520 QTOF LC-MS/MS		
Analytical-scale HPLC		

Notes:

All samples were treated with 3 plugs except for LAT P-ID2466 and LAT F-ID625 which were both treated with 2 plugs

All samples treated using Trypsin Gold-Mass Spec Grade V5280

EGG SAMPLES FOR MASS SPEC ANALYSIS 05/10/16 Stella Chang

Count	Spot ID	Images	Score
1	LAT E-ID104	1	4
2	LAT E-ID105	1	4
3	LAT E-ID112	1	2
4	LAT E-ID113	1	4
5	LAT E-ID116	1	4
6	LAT E-ID121	1	4
7	LAT E-ID128	1	4
8	LAT E-ID129	1	4
9	LAT E-ID157	1	1
10	LAT E-ID183	1	1
11	LAT E-ID252	1	4
12	LAT E-ID257	1	4
13	LAT E-ID267	1	4
14	LAT E-ID268	1	4
15	LAT E-ID276	1	4
16	LAT E-ID277	1	4
17	LAT E-ID281	1	4
18	LAT E-ID282	1	4
19	LAT E-ID291	1	4
20	LAT E-ID293	1	4
21	LAT E-ID307	1	4
22	LAT E-ID308	1	4
23	LAT E-ID312	1	4
24	LAT E-ID313	1	4
25	LAT E-ID316	1	4
26	LAT E-ID317	1	4
27	LAT E-ID322	1	4
28	LAT E-ID326	1	4
29	LAT E-ID327	1	4
30	LAT E-ID366	1	4
31	LAT E-ID564	1	4
32	LAT E-ID628	1	4
33	LAT E-ID633	1	4
34	LAT E-ID640	1	4
35	LAT E-ID643	1	3
36	LAT E-ID646	1	4
37	LAT E-ID650	1	4
38	LAT E-ID685	1	3
39	LAT E-ID98	1	4

LARVA SAMPLES FOR MASS SPEC ANALYSIS 05/10/16 Stella Chang

Count	Spot ID	Images	Score
1	LAT L-ID130	2	4
2	LAT L-ID136	2	2
3	LAT L-ID372	2	4
4	LAT L-ID379	2	4
5	LAT L-ID395	2	4
6	LAT L-ID401	2	1
7	LAT L-ID407	2	2
8	LAT L-ID416	2	1
9	LAT L-ID419	2	4
10	LAT L-ID426	2	1
11	LAT L-ID454	2	2
12	LAT L-ID485	2	2
13	LAT L-ID551	2	4
14	LAT L-ID566	2	1
15	LAT L-ID571	2	1
16	LAT L-ID59	2	4
17	LAT L-ID674	2	1
18	LAT L-ID679	2	2
19	LAT L-ID708	2	4
20	LAT L-ID710	2	3
21	LAT L-ID721	2	1
22	LAT L-ID739	2	4
23	LAT L-ID2001	3	4
24	LAT L-ID2120	3	4
25	LAT L-ID2187	3	4
26	LAT L-ID2367	3	1
27	LAT L-ID2483	3	3
28	LAT L-ID2487	3	4
29	LAT L-ID2496	3	4
30	LAT L-ID2569	3	4
31	LAT L-ID2590	3	1
32	LAT L-ID2634	3	4
33	LAT L-ID2652	3	2

PUPA SAMPLES FOR MASS SPEC ANALYSIS 05/10/16 Stella Chang

Count	Spot ID	Images	Score
1	LAT P-ID2070	4	4
2	LAT P-ID2074	4	1
3	LAT P-ID2262	4	4
4	LAT P-ID2304	4	2
5	LAT P-ID2305	4	4
6	LAT P-ID2322	4	4
7	LAT P-ID2435	4	4
8	LAT P-ID2436	4	4
9	LAT P-ID2466	4	3
10	LAT P-ID2558	4	1
11	LAT P-ID211	5	1
12	LAT P-ID500	5	4
13	LAT P-ID519	5	4
14	LAT P-ID1511	5	4
15	LAT P-ID1654	5	4

FEMALE SAMPLES FOR MASS SPEC ANALYSIS 05/10/16 Stella Chang

Count	Spot ID	Images	Score
1	LAT F-ID223	8	4
2	LAT F-ID226	8	3
3	LAT F-ID299	8	4
4	LAT F-ID363	8	1
5	LAT F-ID403	8	4
6	LAT F-ID533	8	4
7	LAT F-ID541	8	4
8	LAT F-ID548	8	3
9	LAT F-ID625	8	1
10	LAT F-ID654	8	1
11	LAT F-ID779	8	3
12	LAT F-ID1051	9	1
13	LAT F-ID1253	9	4
14	LAT F-ID903	9	1
15	LAT F-ID911	9	4
16	LAT F-ID972	9	4
17	LAT F-ID978	9	3

MALE SAMPLES FOR MASS SPEC ANALYSIS 05/10/16 Stella Chang

Count	Spot ID	Images	Score
1	LAT M-ID1251	6	4
2	LAT M-ID1261	6	4
3	LAT M-ID1288	6	1
4	LAT M-ID1344	6	1
5	LAT M-ID1535	6	4
6	LAT M-ID1617	6	1
7	LAT M-ID1475	7	4
8	LAT M-ID1561	7	4

Egg - Image 1

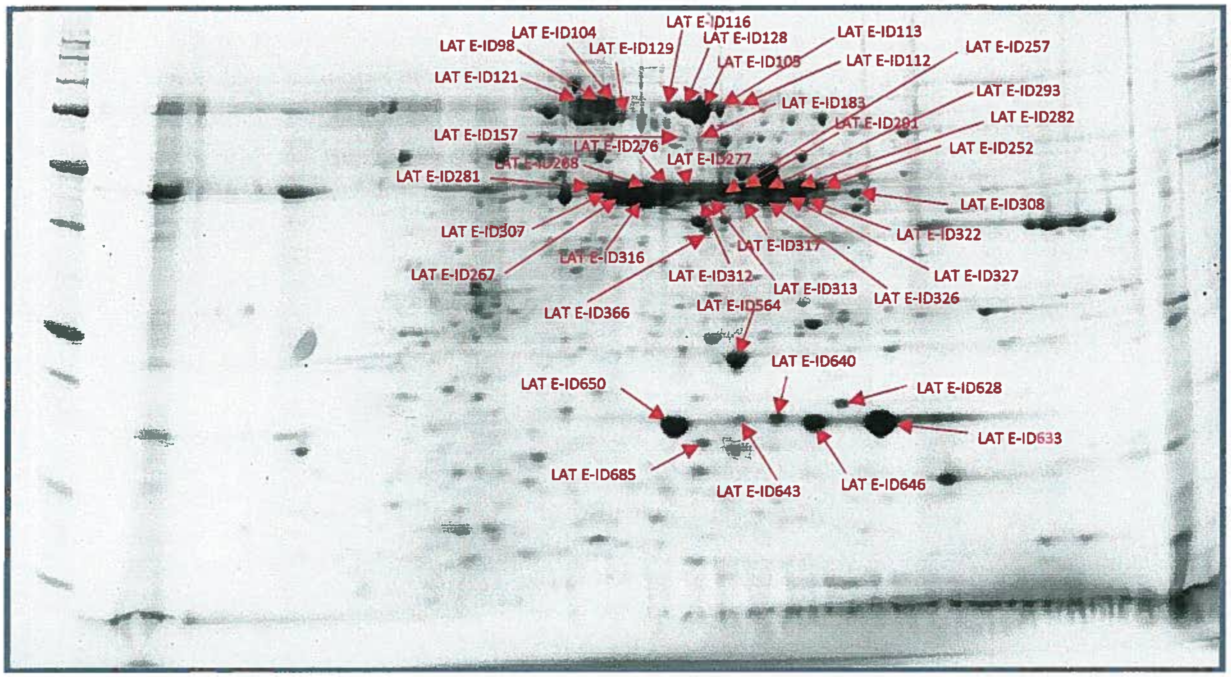

Larva- Image 2

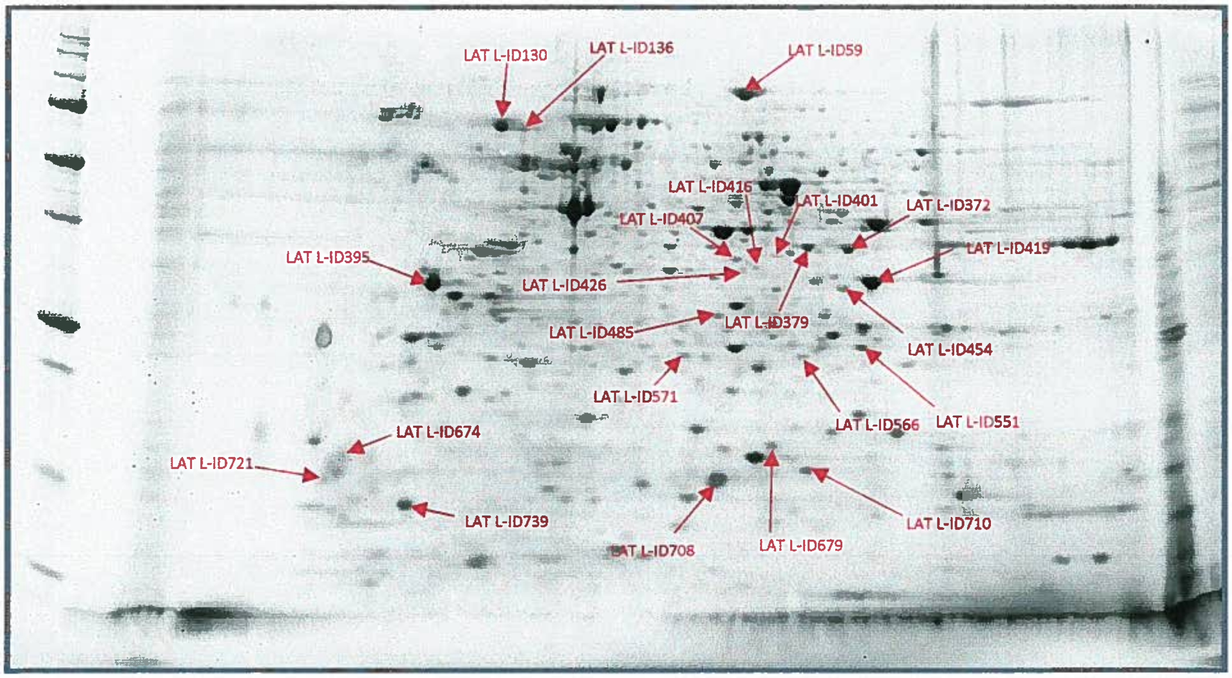

Larva- Image 3

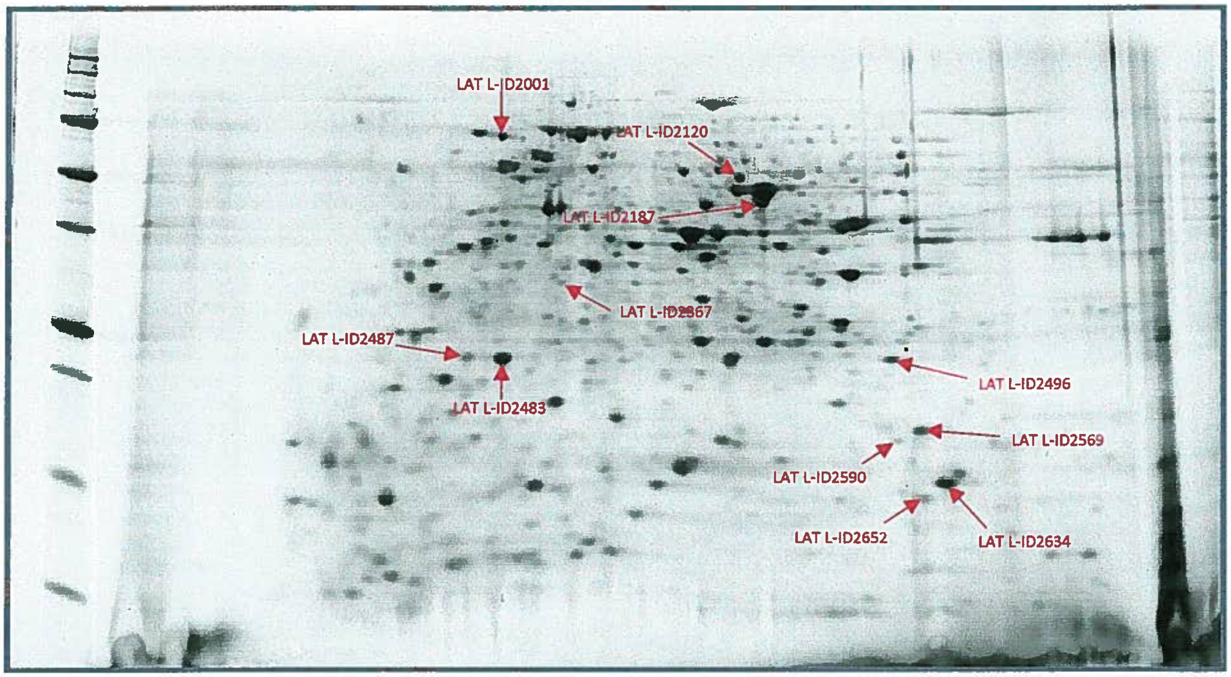

Pupa- Image 4

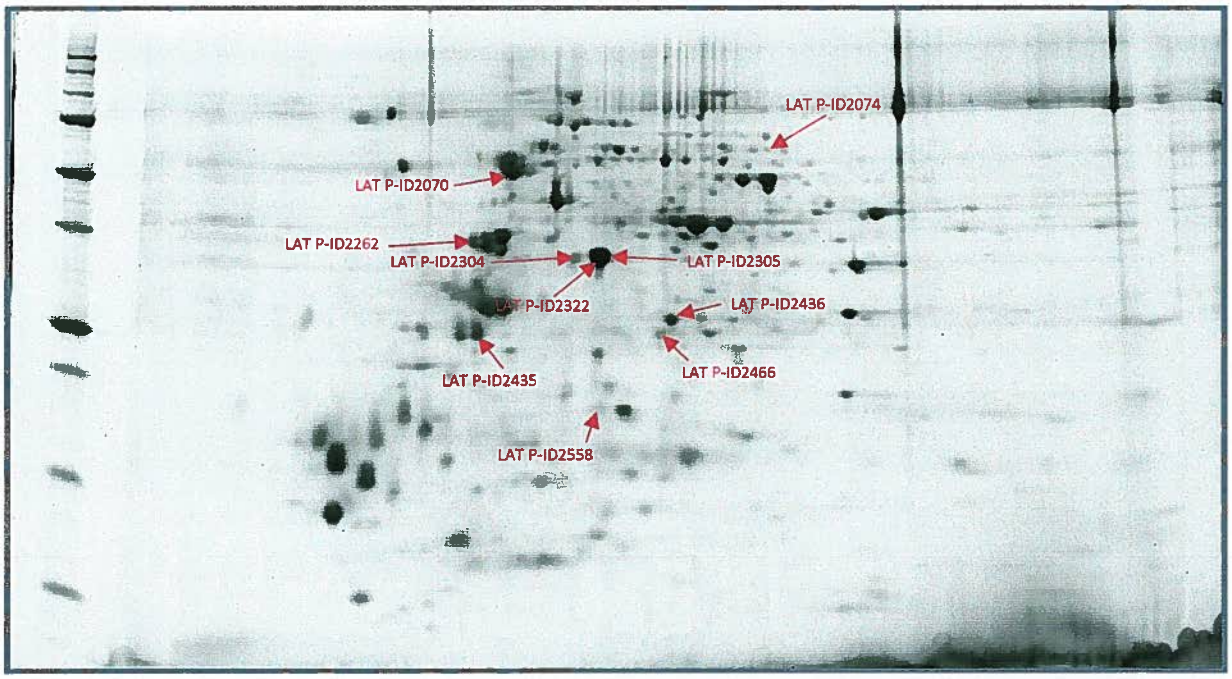

Pupa- Image 5

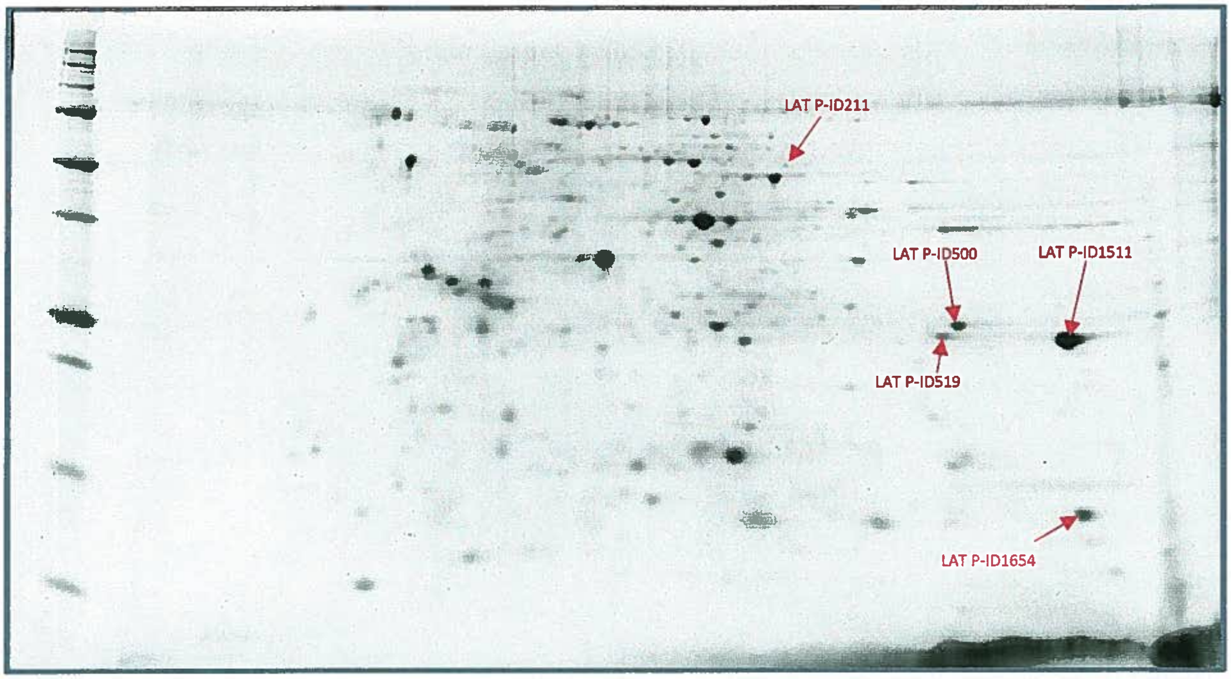

Male- Image 6

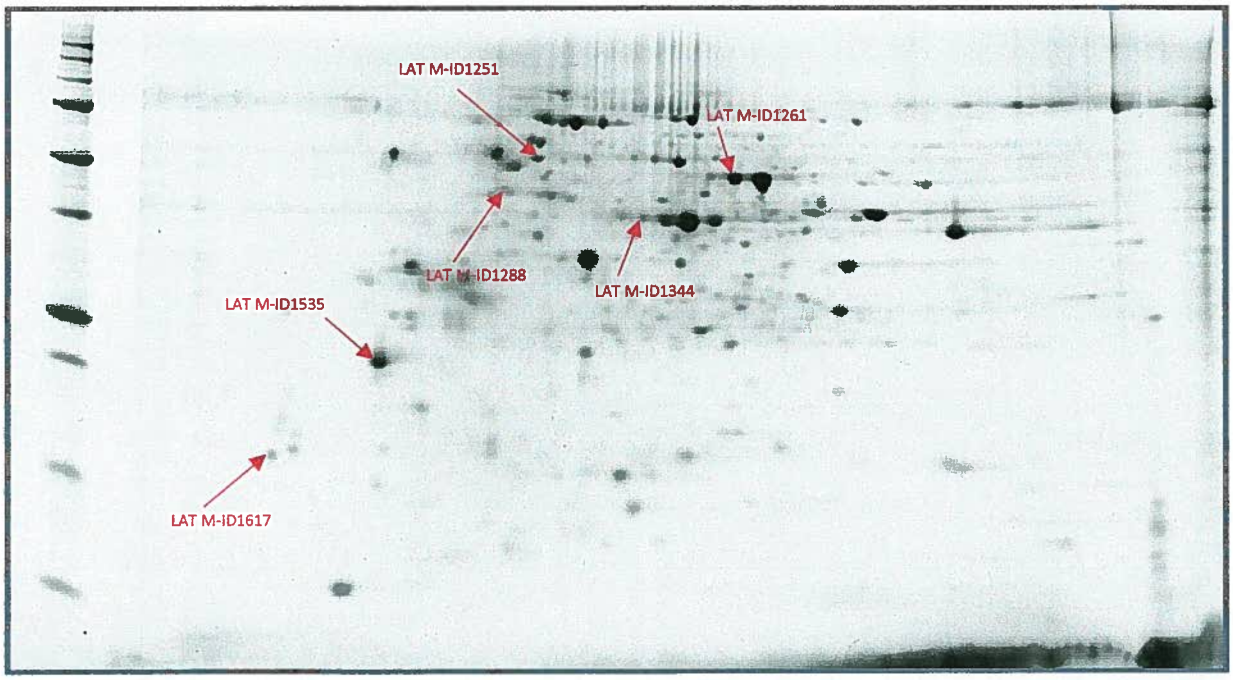

Male- Image 7

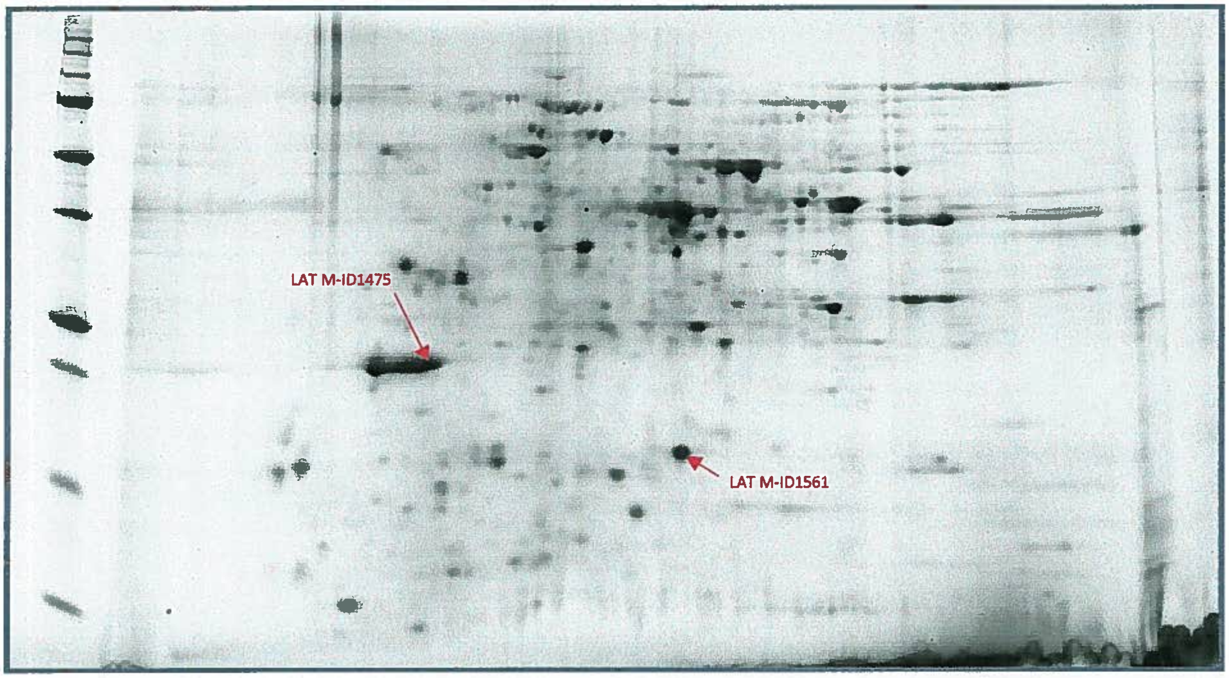

Female- Image 8

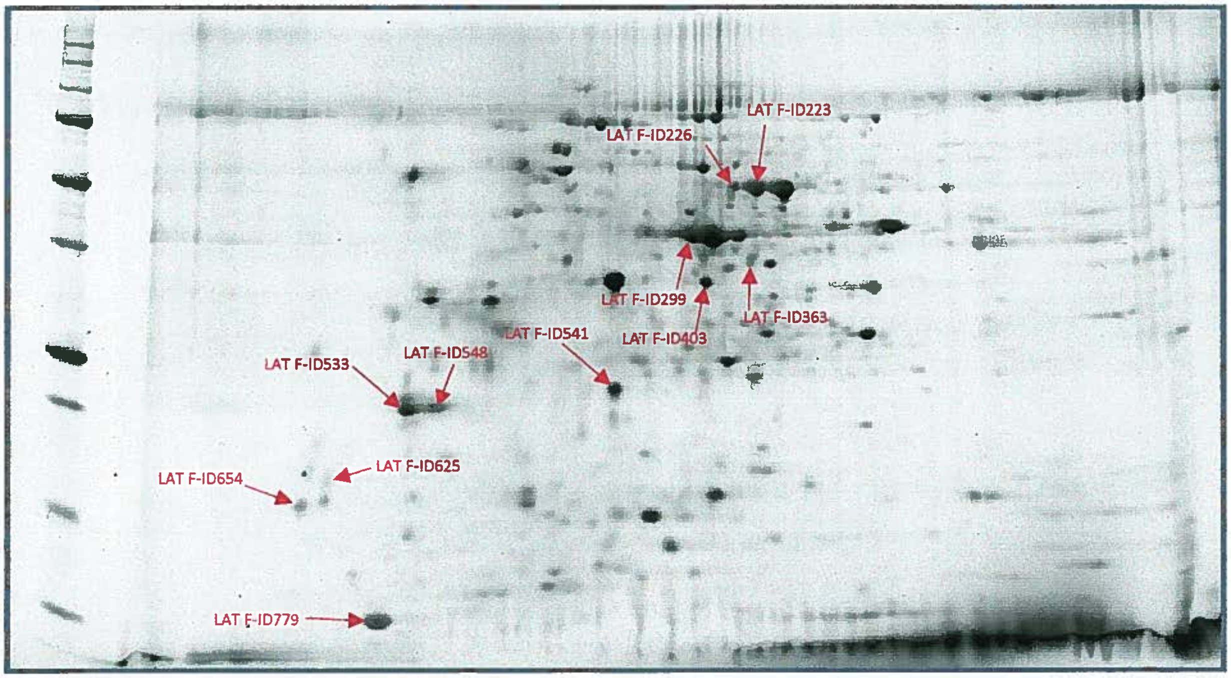

Female- Image 9

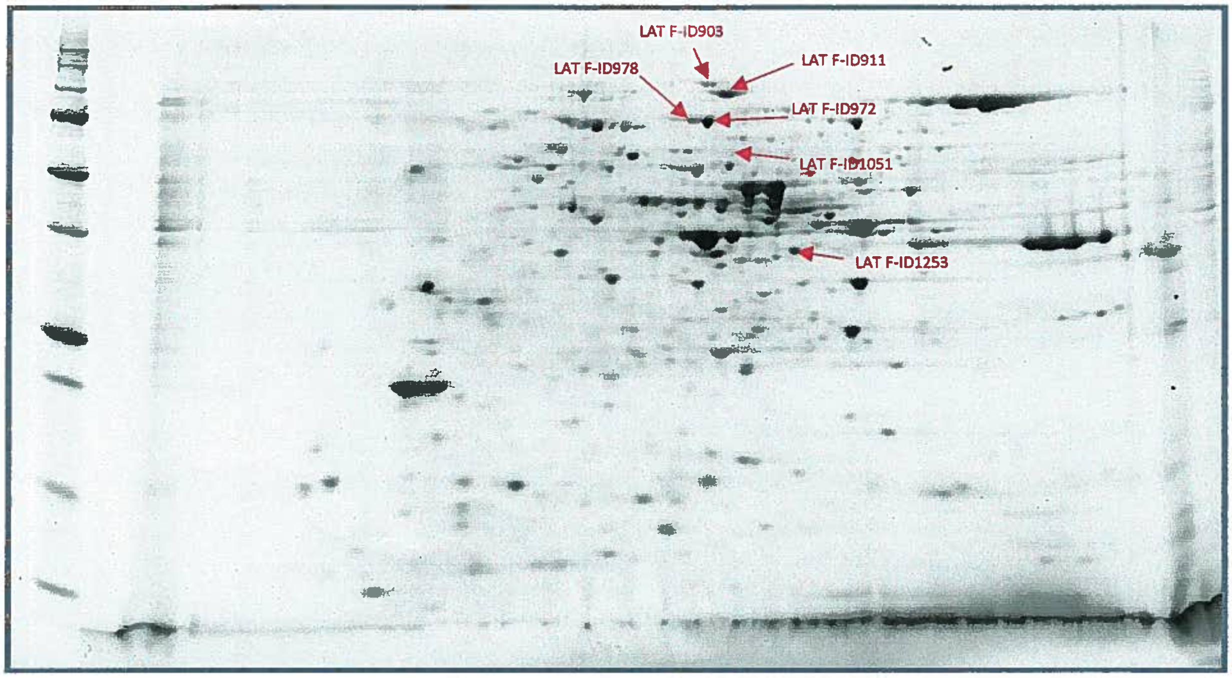

Supplement: Supplementary file 1 [file ijms-19-01996-s001.zip › Supplementary file 1.pdf]
