# Supplementary material for: Comparative Proteomic Profiling between Each of Two Consecutive Developmental Stages of the Solanum Fruit Fly, Bactrocera latifrons (Hendel)
Source: Int J Mol Sci. 2018 Jul 9;19(7):1996. doi: 10.3390/ijms19071996 (PMC6073878; doi:10.3390/ijms19071996)
Supplement: Supplementary file 1 [file ijms-19-01996-s001.zip › Supplementary file 2.pdf]

Stage-ag	Accession number	MW	pI	PS	Ion S	No.	% p	E Value	Peptide Sequence (with highest ion score)	Protein names	Ratio*	SE	p-value
E3-104	XP_018793231.1	75905	5.52	430	278	48	61	1.50E-36	ELNDDNSIITVINIGPR (83)	Maltase 2-like isoform X1	0.11	6.37	0.0000
E3-105	XP_018784870.1	72440	6.29	955	636	53	82	4.70E-89	AVTEYHTCNLEANLPSAVFIR (149)	Transferrin	0.04	6.91	0.0006
E3-112	XP_018784870.1	72440	6.29	799	559	38	69	1.90E-73	AVTEYHTCNLEANLPSAVFIR (118)	Transferrin	0.24	0.47	0.0000
E3-113	XP_018784870.1	72440	6.29	774	533	54	77	5.90E-71	AVTEYHTCNLEANLPSAVFIR (128)	Transferrin	0.07	2.54	0.0001
E3-116	XP_018784870.1	72440	6.29	774	580	50	74	5.90E-71	AVTEYHTCNLEANLPSAVFIR (146)	Transferrin	0.12	2.37	0.0008
E3-121	XP_018793231.1	75905	5.52	524	436	57	65	5.90E-46	ELNDDNSIITVINIGPR (142)	Maltase 2-like isoform X1	0.28	3.00	0.0009
E3-128	XP_018784870.1	72440	6.29	928	614	46	77	2.40E-86	NADLVVVPGNQYVDAR (124)	Transferrin	0.04	7.90	0.0004
E3-129	XP_018793231.1	75905	5.52	564	443	52	54	5.90E-50	ELNDDNSIITVINIGPR (138)	Maltase 2-like isoform X1	0.35	0.66	0.0019
E3-149	XP_018793231.1	75905	5.52	122	102	34	50	9.40E-06	ELNDDNSIITVINIGPR (60)	Maltase 2-like isoform X1	0.05	2.76	0.0004
E3-157	XP_018784870.1	72440	6.29	426	328	31	54	3.70E-36	AVTEYHTCNLEANLPSAVFIR (99)	Transferrin	0.44	0.47	0.0011
E3-183	XP_018784870.1	72440	6.29	539	441	38	72	1.90E-47	AVTEYHTCNLEANLPSAVFIR (98)	Transferrin	0.26	1.18	0.0015
E3-252	XP_018798053.1	48195	8.64	327	226	33	64	3.00E-26	YYAESVRPGNEHNFAAVPASSK (85)	Vitellogenin-1-like	0.26	0.85	0.0005
E3-257	XP_018798053.1	48195	8.64	279	205	33	68	1.90E-21	YYAESVRPGNEHNFAAVPASSK (66)	Vitellogenin-1-like	0.36	1.97	0.0037
E3-267	XP_018787380.1	48061	6.48	553	432	41	67	7.40E-49	NFPAVGANSIDQYENNDGAGKR (159)	Vitellogenin-1	0.14	6.34	0.0000
E3-268	XP_018787380.1	48061	6.48	545	446	33	66	4.70E-48	NFPAVGANSIDQYENNDGAGKR (165)	Vitellogenin-1	0.07	0.98	0.0000
E3-276	XP_018787380.1	48061	6.48	540	412	46	72	1.50E-47	NFPAVGANSIDQYENNDGAGKR (125)	Vitellogenin-1	0.25	1.10	0.0001
E3-277	XP_018787380.1	48061	6.48	344	255	39	74	5.90E-28	NFPAVGANSIDQYENNDGAGKR (117)	Vitellogenin-1	0.12	1.59	0.0004
E3-281	XP_018787380.1	48061	6.48	419	353	34	64	1.90E-35	GDADFVDAIHTSTCGM1GTR (121)	Vitellogenin-1	0.25	2.53	0.0026
E3-282	XP_018798053.1	48195	8.64	432	256	52	85	9.40E-37	QGSYHGLHQAWR (80)	Vitellogenin-1-like	0.15	1.13	0.0000
E3-291	XP_018798053.1	48195	8.64	340	178	45	80	1.50E-27	QGSYHGLHQAWR (80)	Vitellogenin-1-like	0.34	1.19	0.0009
E3-293	XP_018798053.1	48195	8.64	298	156	41	71	2.40E-23	QGSYHGLHQAWR (64)	Vitellogenin-1-like	0.09	1.37	0.0000
E3-307	XP_018787380.1	48061	6.48	434	309	40	62	5.90E-37	NFPAVGANSIDQYENNDGAGKR (133)	Vitellogenin-1	0.17	2.85	0.0021
E3-308	XP_018787290.1	46162	6.62	219	152	31	69	1.90E-15	GNADFVDAIHTSAYGLGSVAR (126)	Vitellogenin-2-like	0.17	5.79	0.0005
E3-312	XP_018787290.1	46162	6.62	389	353	26	65	1.90E-32	GNADFVDAIHTSAYGLGSVAR (127)	Vitellogenin-2-like	0.11	9.88	0.0019
E3-313	XP_018787290.1	46162	6.62	295	266	24	65	4.70E-23	GNADFVDAIHTSAYGLGSVAR (119)	Vitellogenin-2-like	0.39	0.57	0.0013
E3-316	XP_018787380.1	48061	6.48	517	376	48	77	3.00E-45	NFPAVGANSIDQYENNDGAGKR (131)	Vitellogenin-1	0.10	1.65	0.0002
E3-317	XP_018787290.1	46162	6.62	493	453	28	73	7.40E-43	GNADFVDAIHTSAYGLGSVAR (138)	Vitellogenin-2-like	0.46	1.03	0.0017
E3-322	XP_018787290.1	46162	6.62	410	352	29	75	1.50E-34	GNADFVDAIHTSAYGLGSVAR (142)	Vitellogenin-2-like	0.39	0.45	0.0002
E3-326	XP_018787290.1	46162	6.62	555	532	24	61	4.70E-49	GNADFVDAIHTSAYGLGSVAR (137)	Vitellogenin-2-like	0.43	1.83	0.0019
E3-327	XP_018787290.1	46162	6.62	378	266	37	70	2.40E-31	GNADFVDAIHTSAYGLGSVAR (150)	Vitellogenin-2-like	0.19	1.65	0.0007
E3-366	XP_018791497.1	36639	6.09	400	253	40	78	1.50E-33	GAYYCVADNGVGQPDER (75)	Protein amalgam	0.32	0.41	0.0050
E3-564	XP_011201883.1	21998	6.09	572	494	26	80	9.40E-51	DYGVLDEETGIPFR (115)	Peroxiredoxin 1	0.27	1.24	0.0000
E3-628	XP_018795769.1	22302	7.77	699	634	29	90	1.90E-63	NFKELAEKPEGEGYKGSK (127)	Peptidyl-prolyl cis-trans isoMerase 5	0.46	0.80	0.0043
E3-633	XP_018788060.1	18433	7.11	344	309	22	82	5.90E-28	DGFQVSM1DVAHFKPSELNVR (132)	Heat shock protein 23-like	0.16	1.85	0.0000
E3-640	XP_018788060.1	18433	7.11	320	311	15	77	1.50E-25	DGFQVSM1DVAHFKPSELNVR (131)	Heat shock protein 23-like	0.12	2.64	0.0001
E3-643	XP_018788060.1	18433	7.11	232	271	14	73	9.40E-17	DGFQVSM1DVAHFKPSELNVR (79)	Heat shock protein 23-like	0.08	3.13	0.0000
E3-646	XP_018788060.1	18433	7.11	364	356	18	78	5.90E-30	DGFQVSM1DVAHFKPSELNVR (142)	Heat shock protein 23-like	0.19	3.01	0.0008
E3-650	XP_018788062.1	19058	5.96	244	220	13	82	5.90E-18	VVQIQQTGPAHLNVKENPK (70)	Heat shock protein 23-like	0.23	1.06	0.0000
E3-685	XP_018797702.1	20084	8.69	353	279	21	87	7.40E-29	SLELTIDLPPLGGLR (99)	Peroxiredoxin-5, Mitochondrial	0.49	1.58	0.0022
E3-709	XP_018799095.1	17076	5.10	309	225	24	69	1.90E-24	RKENNYPDVESVR (50)	General odorant-binding protein 99a-like	0.25	0.38	0.0000
E3-98	XP_018793231.1	75905	5.52	726	553	59	67	3.70E-66	ELNDDNSIITVINIGPR (145)	Maltase 2-like isoform X1	0.25	1.41	0.0001
L1-130	XP_018793742.1	62297	4.97	308	252	27	38	2.40E-24	EIPGETINLQTCVR (94)	Uncharacterized protein LOC108971874	4.37	0.07	0.0003
L1-136	XP_018793741.1	61607	5.00	363	254	32	51	7.40E-30	GQPYCSLPNACPLTTR (111)	Uncharacterized protein LOC108971872 iso	5.89	0.15	0.0014
L1-304	XP_004526477.1	42118	5.23	877	669	44	81	3.00E-81	TTGIVLDSGDGVSHTVPIYEGYALPHAIL	Actin-2, muscle-specific	3.82	0.37	0.0000
L1-365	XP_018799378.1	33064	4.82	406	308	38	72	3.70E-34	KLAFVEDELEVAEDRVR (86)	Tropomyosin-2 isoform X2	20.31	0.03	0.0001
L1-372	XP_018802321.1	36231	6.99	428	304	27	44	2.40E-36	LCIVTAGVR (63)	L-lactate dehydrogenase	2.67	2.04	0.0044
L1-378	XP_018799300.1	32898	4.71	851	673	42	90	1.20E-78	ALQNAESEVAALNR (117)	TropoMyosin-2 isoform X20	7.12	0.08	0.0000
L1-379	XP_018783435.1	35904	6.67	350	218	31	60	1.50E-28	AQGQAIDQHAR (68)	Malate dehydrogenase, cytoplasmic	2.62	0.27	0.0023
L1-395	XP_018795679.1	39452	4.66	529	363	22	86	1.90E-46	KSELLAGLNSEGR (127)	Uncharacterized protein LOC108973107 iso	5.17	0.13	0.0000
L1-401	XP_018789491.1	39902	6.51	178	99	24	59	2.40E-11	FLPVTEEHPTGNCTSPYGK (40)	UDP-glucose 4-epimerase	5.13	0.43	0.0141
L1-407	XP_018804575.1	39830	7.04	300	205	26	52	1.50E-23	NTPSYQSILENANVLAR (91)	Fructose-bisphosphate aldolase	6.18	0.15	0.0023
L1-416	XP_018787334.1	35757	8.26	356	282	18	72	3.70E-29	VPTPNVSVVDLTVR (113)	Glyceraldehyde-3-phosphate dehydrogenase	4.45	0.08	0.0006
L1-419	XP_018804543.1	31185	7.11	737	506	52	94	3.00E-67	GLIQGHGFQLIETPYALEGKPR (154)	Trehalose-phosphate phosphatase B	15.82	0.18	0.0001
L1-426	XP_004519608.1	59908	6.51	93	24	20	34	7.80E-03	TLAQNCGANTIR (22)	T-complex protein 1 subunit gamma	23.12	0.09	0.0010
L1-454	XP_018804039.1	35059	8.53	298	186	19	68	2.40E-23	QGLDPHVFAEIINSSTGR (75)	Probable 3-hydroxyisobutyrate dehydrogena	2.14	0.76	0.0006
L1-485	XP_018804575.1	39830	7.04	216	124	21	52	3.70E-15	NTPSYQSILENANVLAR (94)	Fructose-bisphosphate aldolase	2.01	0.45	0.0268
L1-551	XP_018804543.1	31185	7.11	441	326	35	83	1.20E-37	GLIQGHGFQLIETPYALEGKPR (119)	Trehalose-phosphate phosphatase B	2.04	0.89	0.0024
L1-563	XP_018794827.1	27527	5.13	606	418	29	75	3.70E-54	TTILDGLTGTFPEEFNQYLHK (153)	Uncharacterized protein LOC108972624	3.30	0.17	0.0422
L1-565	XP_018794827.1	27527	5.13	972	671	36	83	9.40E-91	TTILDGLTGTFPEEFNQYLHK (129)	Uncharacterized protein LOC108972624	26.86	0.04	0.0007
L1-566	XP_011210145.1	25805	7.53	309	246	15	55	1.90E-24	SQGDWSPSGGIGLPQPDNR (88)	Uncharacterized protein LOC105230849	6.57	0.14	0.0006
L1-571	XP_018793665.1	21623	5.95	362	252	18	81	9.40E-30	LLDQHFGTGLR (68)	Protein lethal(2)essential for life	2.13	0.89	0.0017
L1-59	XP_018791794.1	101121	5.91	820	551	73	71	1.50E-75	ALNASIQAPSGLEEPCFLKR (87)	Filamin-A isoform X2	34.34	0.24	0.0000
L1-674	XP_018787727.1	16173	4.51	184	139	9	43	5.69E-12	TVYYEADENGYR (71)	Larval cuticle protein A3A	15.72	0.33	0.0000
L1-679	XP_018804351.1	20380	8.80	752	609	25	84	9.40E-69	KDIAQVTNTVFALGR (146)	Muscle-specific protein 20	3.02	0.35	0.0018
L1-708	XP_018799091.1	17334	6.44	387	347	17	67	3.00E-32	KLGVFCEHEGYHADR (92)	General odorant-binding protein 99a-like iso	2.01	0.65	0.0004
L1-710	XP_018788026.1	19242	7.85	597	569	18	72	3.00E-53	ITQKHPEYTGPSLGPK (89)	Myophilin	12.10	0.07	0.0000
L1-721	XP_014094514.1	11272	4.28	139	132	2	37	1.90E-07	SDVDPEKYSFALK (75)	Larval cuticle protein 8-like	7.70	0.30	0.0000
L1-739	XP_018797071.1	11722	4.58	511	383	16	82	1.20E-44	ILSSVGIEVDSER (118)	60S acidic ribosomal protein P2	3.64	0.23	0.0015
L1-750	XP_018803514.1	11521	4.24	187	154	6	96	3.00E-12	AANVDVEPYWPGLFAK (105)	60S acidic ribosomal protein P1	3.90	0.39	0.0008
L10-199	XP_018793742.1	62297	4.97	308	252	27	38	2.40E-24	EIPGETINLQTCVR (94)	Uncharacterized protein LOC108971874	0.41	0.89	0.0054
L10-200	XP_018793741.1	61607	5.00	177	90	29	46	3.00E-11	GQPYCSLPNACPLTTR (42)	Uncharacterized protein LOC108971872 iso	0.45	7.61	0.0119
L10-212	XP_018783509.1	52350	6.68	331	201	48	81	1.20E-26	VAFTGSAEIGR (68)	Aldehyde dehydrogenase X, Mitochondrial-	0.50	0.15	0.0082
L10-218	XP_018790778.1	50825	8.87	342	307	30	69	9.40E-28	LGDVYVNDAFGTAHR (98)	Phosphoglycerate kinase	0.34	21.21	0.0019
L10-222	XP_011208284.1	71578	5.42	704	713	33	40	5.90E-64	DNNLLGKFELSGIPPAPR (143)	Heat shock 70 kDa protein cognate 4	0.35	0.59	0.0152
L10-230	XP_018794703.1	47086	4.53	451	344	22	38	1.20E-38	FYSDENVDKGLQTSQDAR (114)	Calreticulin	0.10	1.23	0.0001
L10-232	XP_018795679.1	39452	4.66	529	363	22	86	1.90E-46	KSELLAGLNSEGR (127)	Uncharacterized protein LOC108973107 iso	0.42	0.60	0.0016
L10-236	XP_018789492.1	57690	8.65	251	198	29	59	1.20E-18	TVYEEEEAELTEEEIR (138)	Uncharacterized protein LOC108969313	0.33	0.66	0.0209
L10-245	XP_018802393.1	27184	6.89	658	552	24	78	2.40E-59	VVIVTGASSGIGAITAEEFAK (140)	Uncharacterized protein LOC108977244	0.42	0.61	0.0002
L10-248	XP_018794827.1	27527	5.13	647	432	31	83	3.00E-58	TTILDGLTGTFPEEFNQYLHK (152)	Uncharacterized protein LOC108972624	0.29	0.29	0.0001
L10-248	XP_018794827.1	27527	5.13	393	291	23	75	7.40E-32	TTILDGLTGTFPEEFNQYLHK (117)	Uncharacterized protein LOC108972624	0.34	1.09	0.0032
L10-249	XP_018788990.1	24511	8.64	281	230	14	65	1.20E-21	LPYDYAALEPVICR (88)	Superoxide dismutase [Mn]	0.44	0.31	0.0315
L10-256	XP_018804351.1	20380	8.80	860	726	25	84	1.50E-79	DIAQVTNTVFALGR (140)	Muscle-specific protein 20	0.30	0.20	0.0001
L10-259	XP_018788026.1	19242	7.85	475	451	22	75	4.70E-41	YGVPEEEIFQTADLFER (110)	Myophilin	0.22	0.78	0.0136
L10-261	XP_018804878.1	17277	5.26	333	289	14	94	7.40E-27	LTFAAANQTFYDQSEVR (80)	ATP synthase subunit delta	0.22	0.89	0.0001
L10-263	XP_004517938.2	18812	7.82	324	423	14	48	5.90E-26	QM1LGATNPADSLPGTIR (103)	Nucleoside diphosphate kinase	0.13	2.46	0.0007
L10-265	XP_018804351.1	20380	8.80	627	543	20	70	3.00E-56	KDIAQVTNTVFALGR (149)	Muscle-specific protein 20	0.05	3.37	0.0001
L10-266	XP_018803514.1	11521	4.24	245	297	7	96	4.70E-28	AELACVYAALVLVDDDIAVTGEK (125)	60S acidic ribosomal protein P1	0.42	2.70	0.0068
P12-211	XP_018798316.1	57453	8.36	297	228	35	62	3.00E-23	VNDNQGGAPNYFPNSFSGPDESAR (91)	Catalase	0.30	0.68	0.0133
P12-500	XP_018794196.1	22575	7.85	667	606	9	53	3.00E-60	AAEESLVNGVVHGSYSLTEPDGSIR (114)	Cuticle protein 21	0.03	0.60	0.0000
P12-519	XP_018794197.1	19937	8.01	392	351	8	51	9.40E-33	IVEYAADPVHGFNAVVR (109)	Larval cuticle protein A2B	0.07	3.65	0.0000
P12-522	XP_018794197.1	19937	8.01	395	335	8	51	4.70E-33	IVEYAADPVHGFNAVVR (118)	Larval cuticle protein A2B	0.04	29.38	0.0003
P12-723	XP_018788732.1	14448	6.28	482	424	8	61	9.40E-42	YSFNYGIKDPHTGDIK (96)	Cuticle protein 8	0.10	4.18	0.0026
P1-1481	XP_018794196.1	22575	7.85	667	606	9	53	3.00E-60	AAEESLVNGVVHGSYSLTEPDGSIR (114)	Cuticle protein 21	0.13	2.53	0.0000
