# Supplementary figures and images for: Comparative Proteomic Profiling between Each of Two Consecutive Developmental Stages of the Solanum Fruit Fly, Bactrocera latifrons (Hendel)
Source: Int J Mol Sci. 2018 Jul 9;19(7):1996. doi: 10.3390/ijms19071996 (PMC6073878; doi:10.3390/ijms19071996)

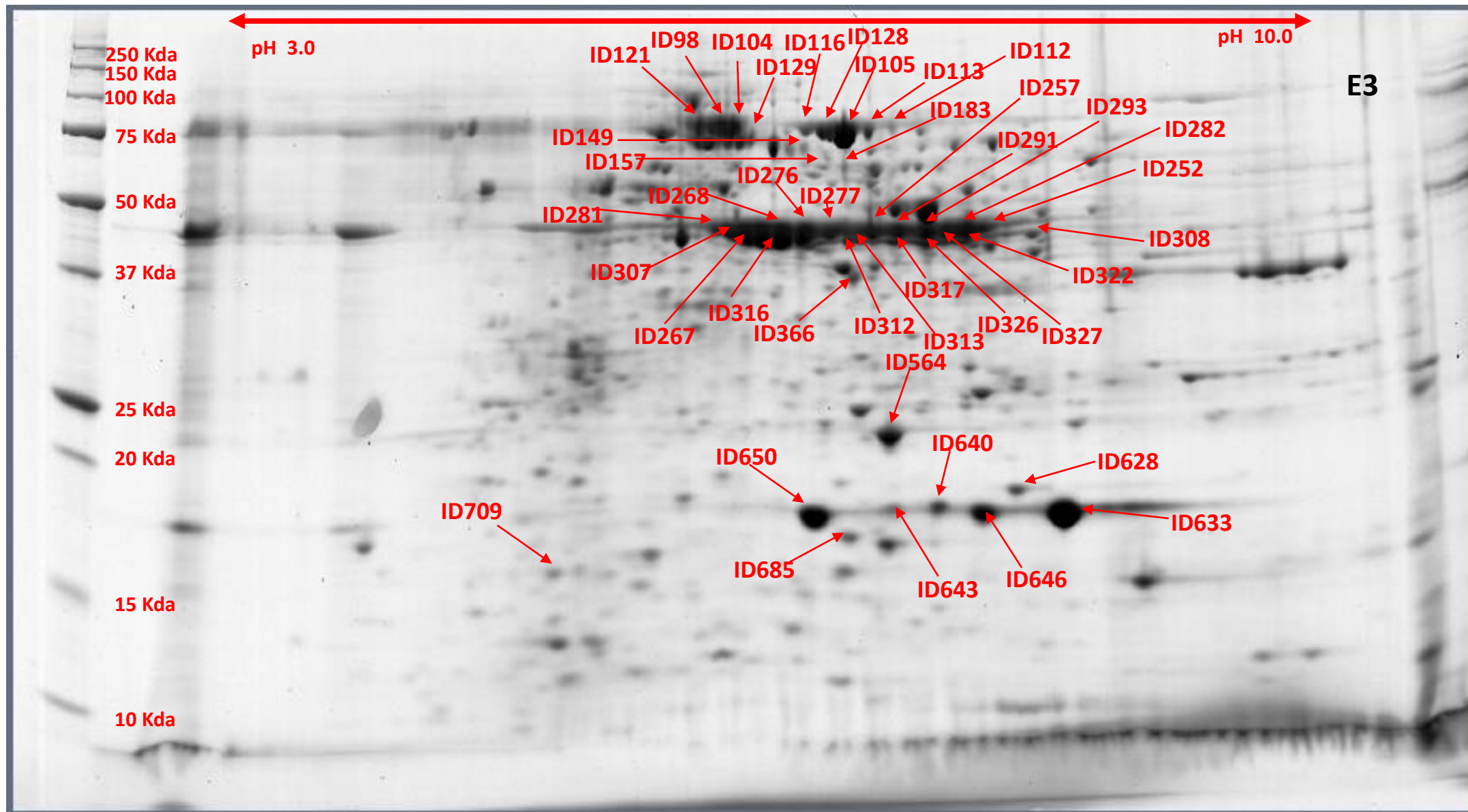

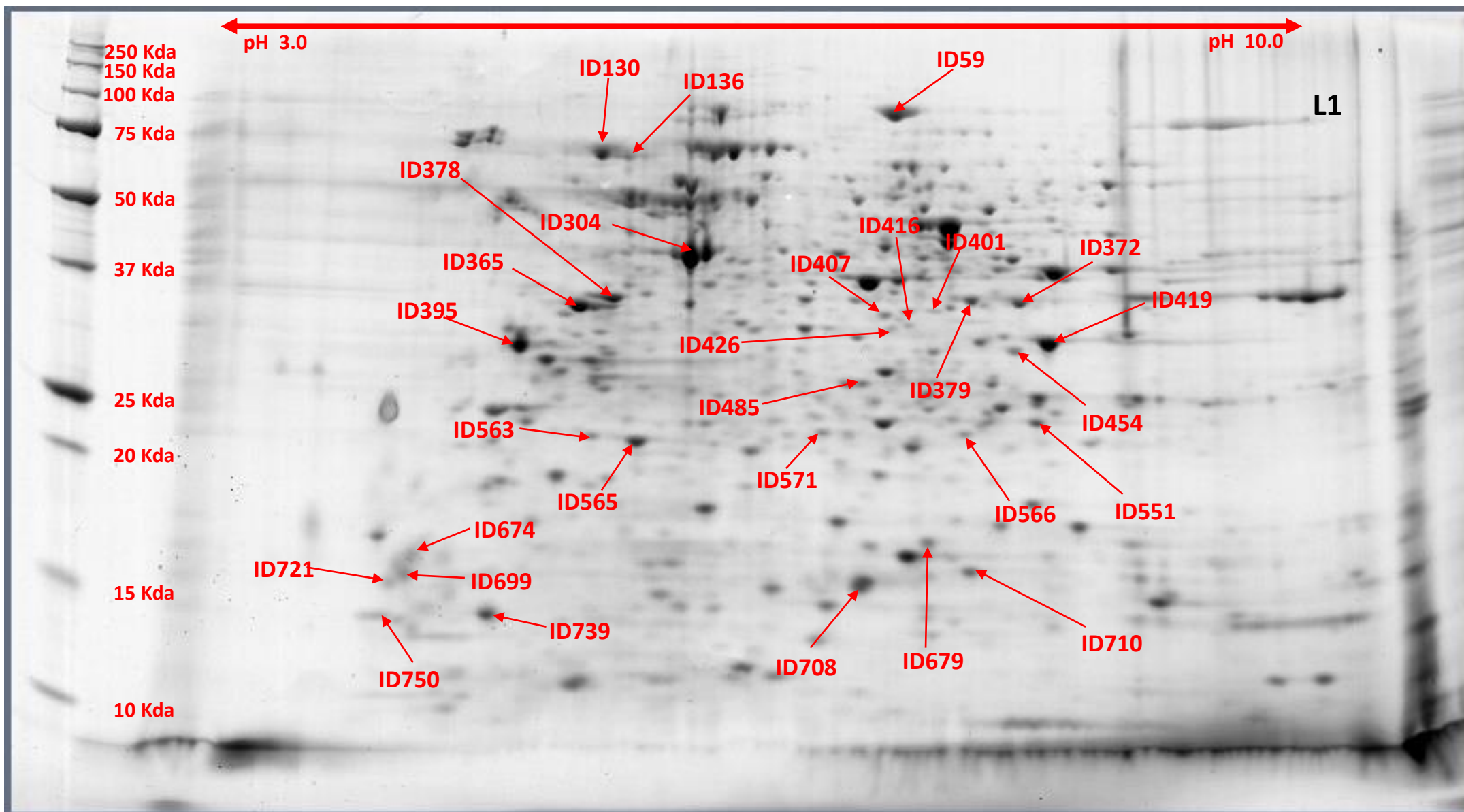

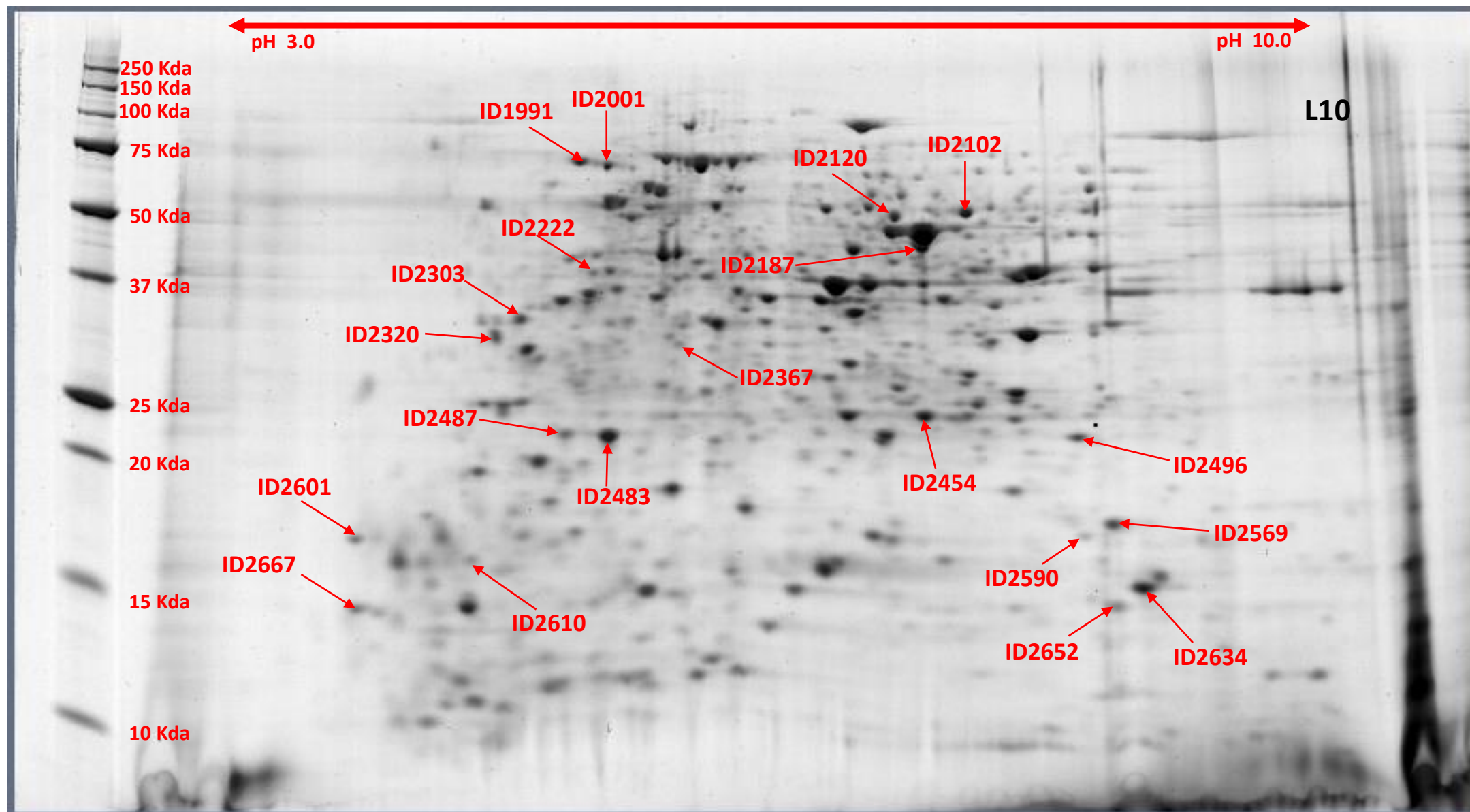

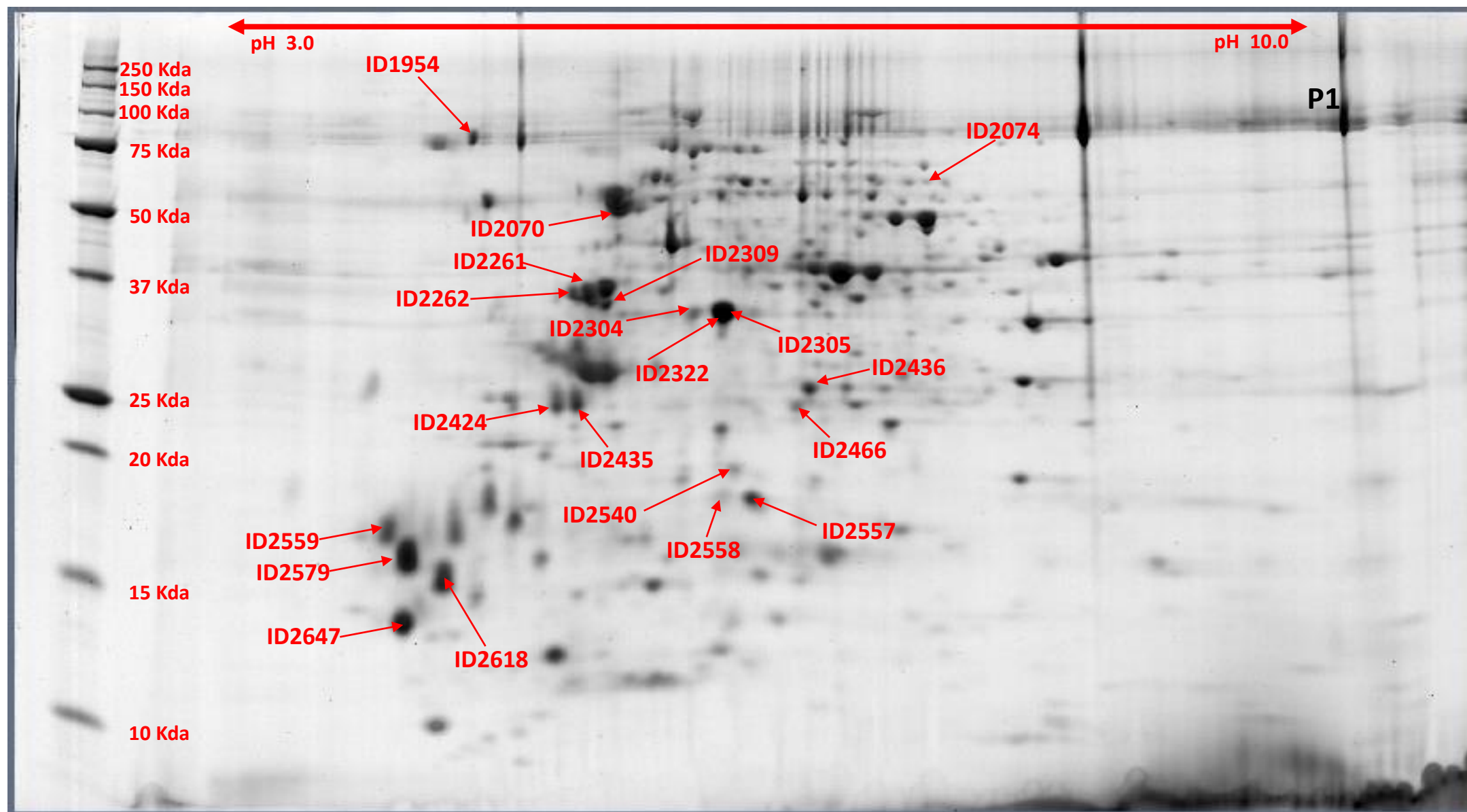

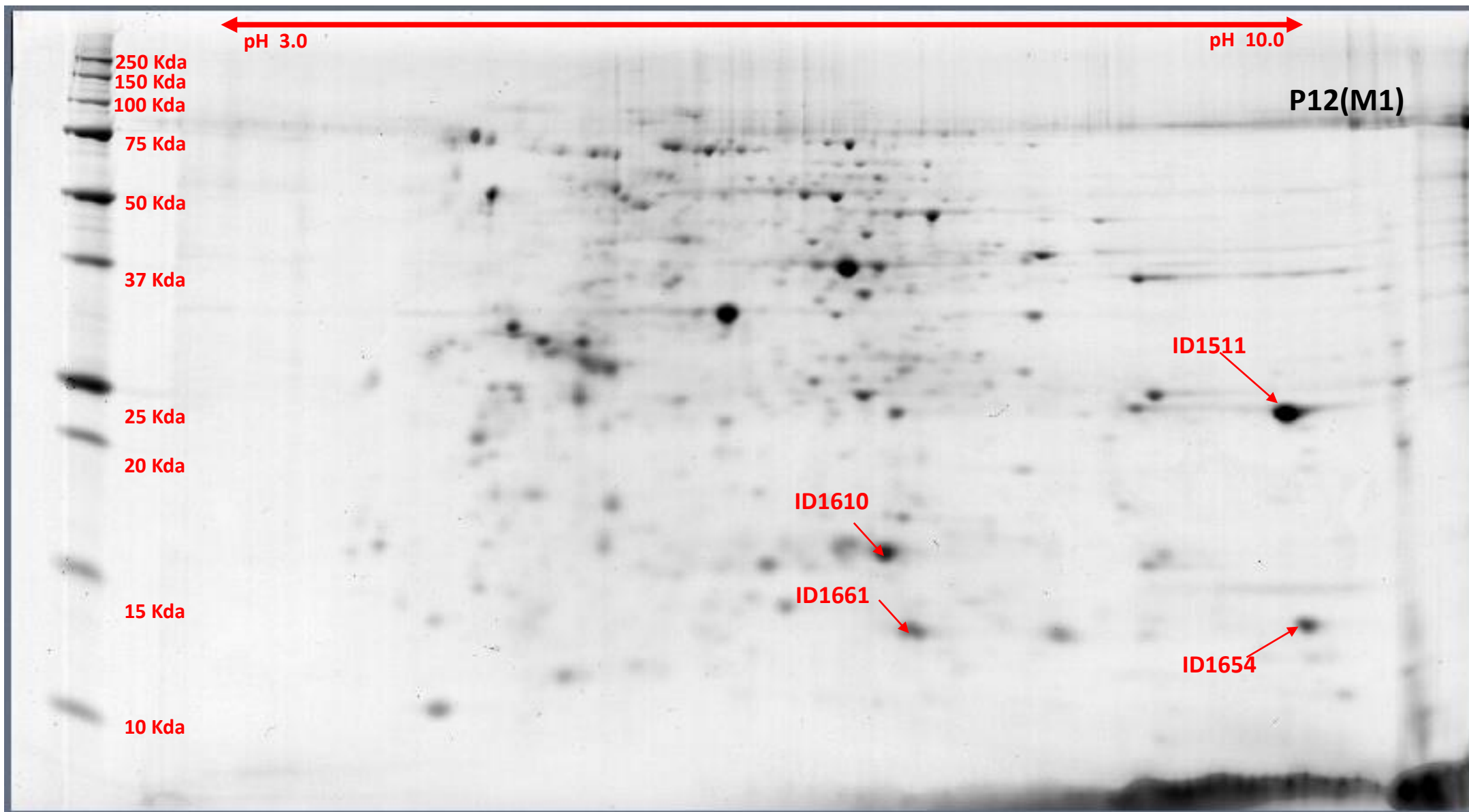

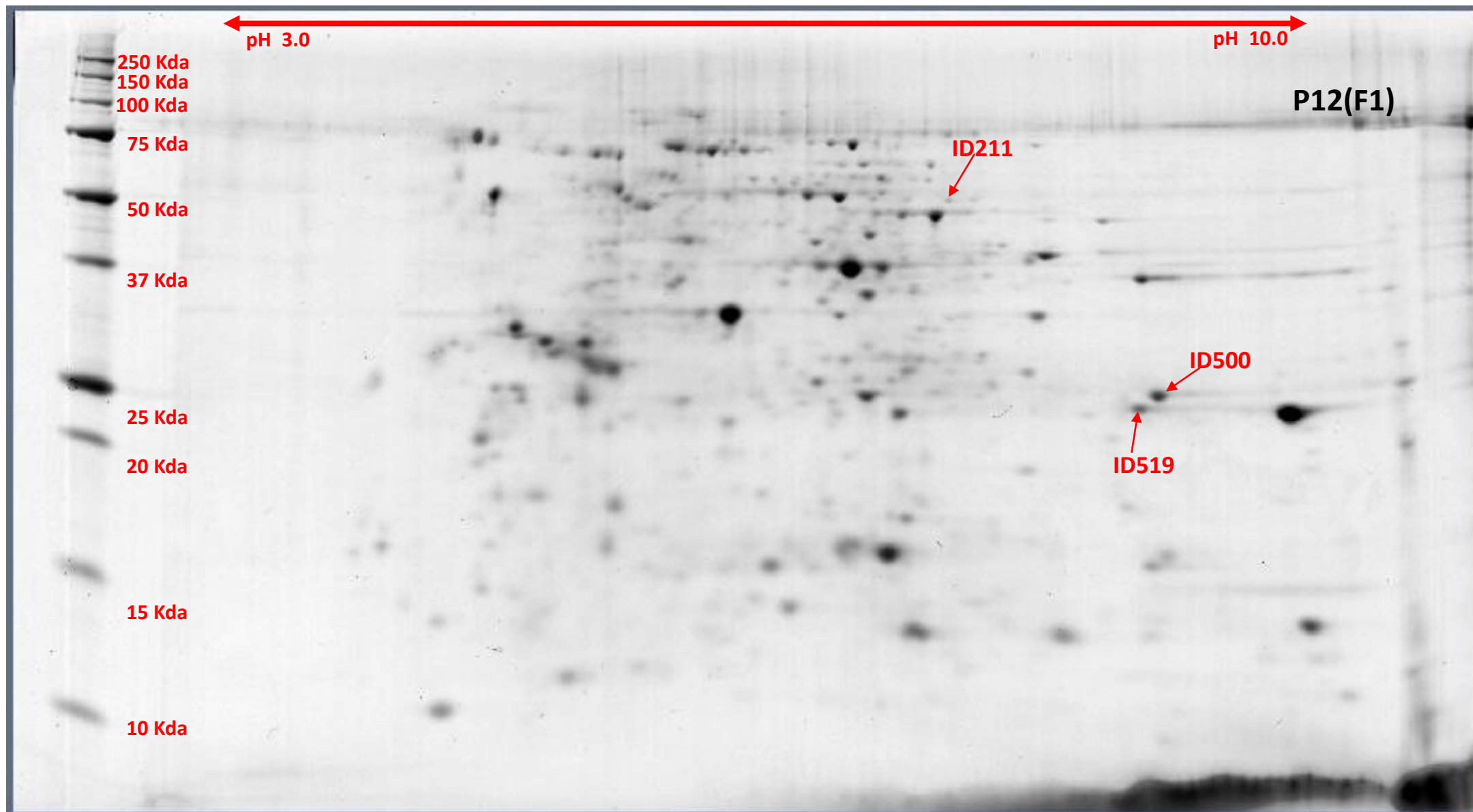

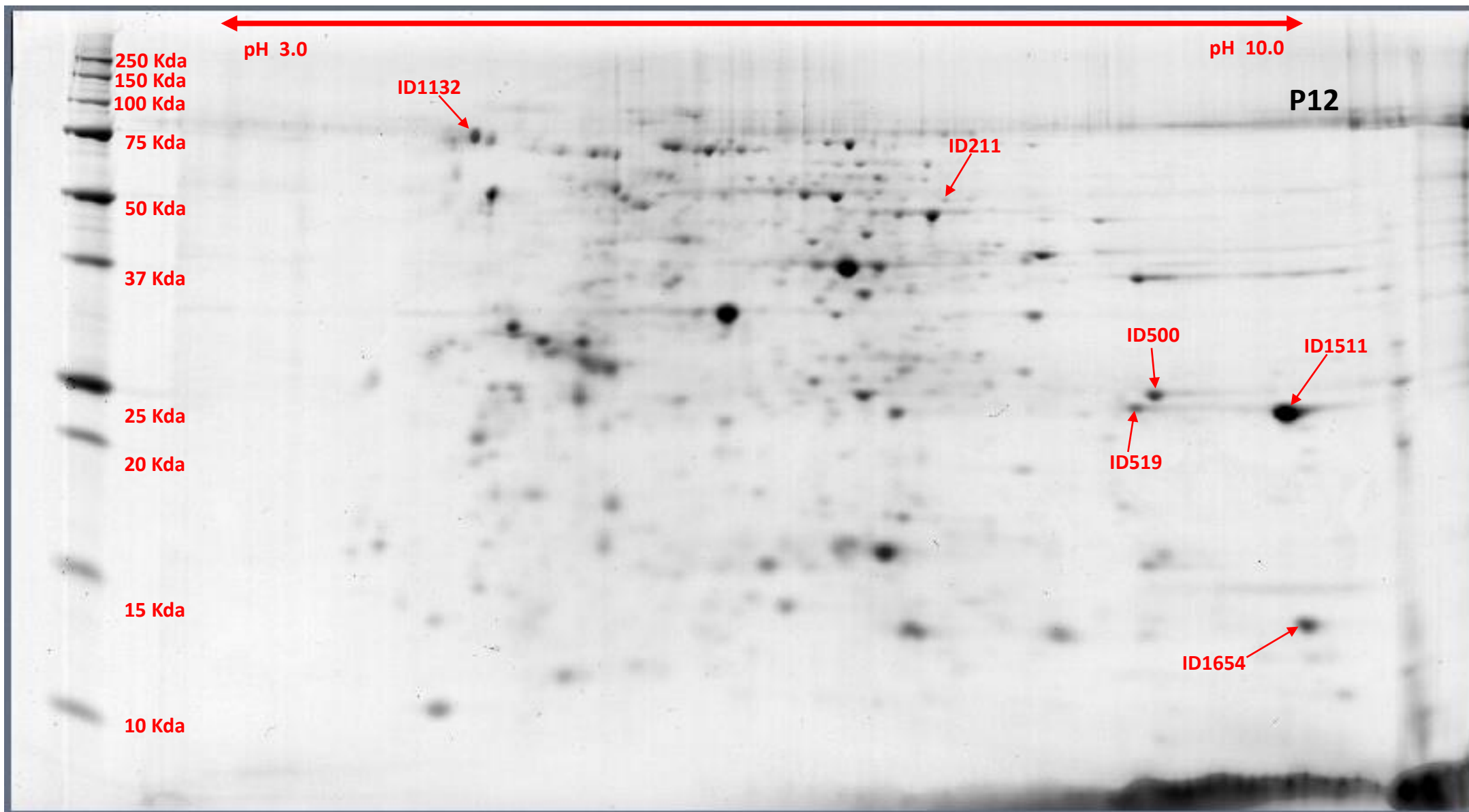

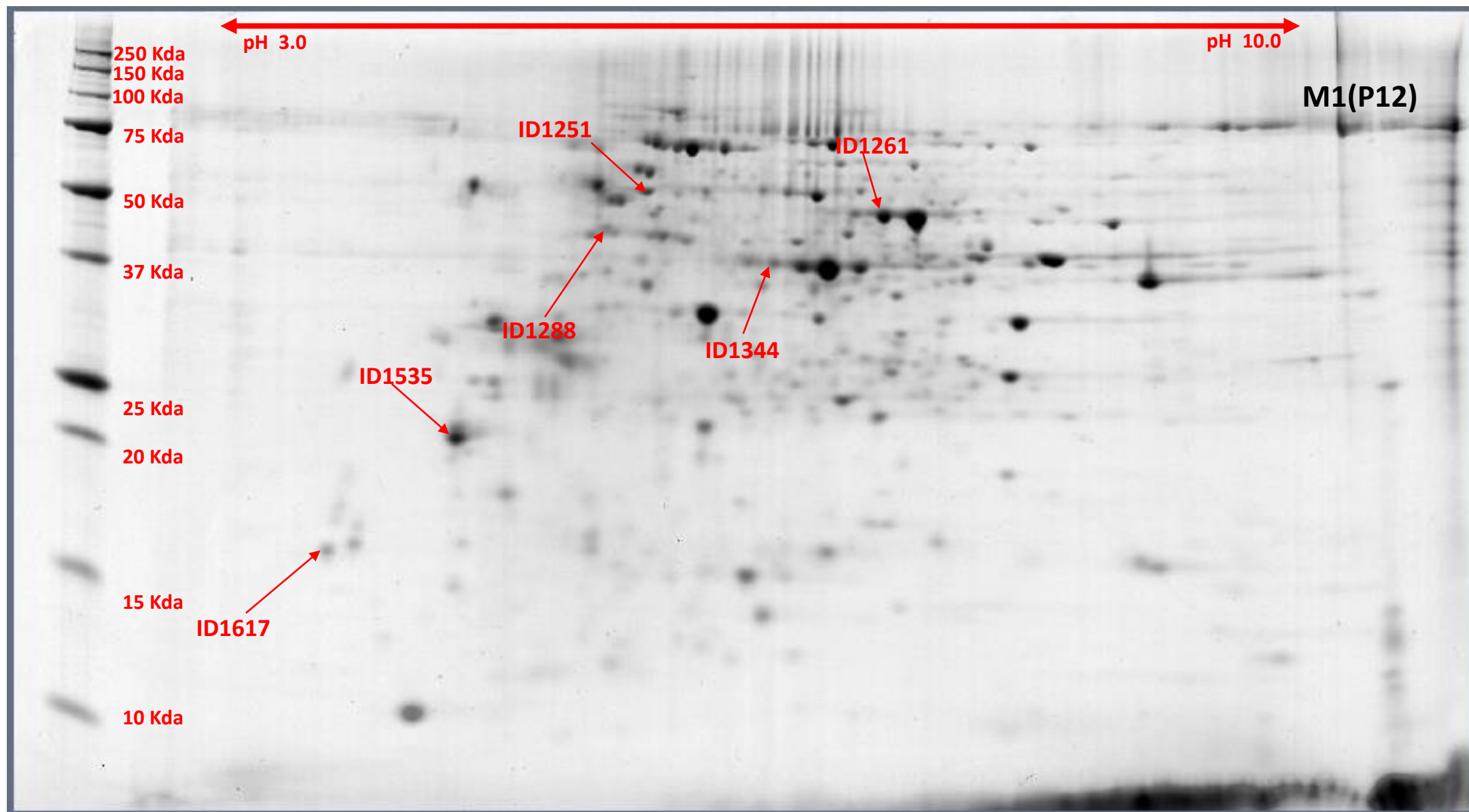

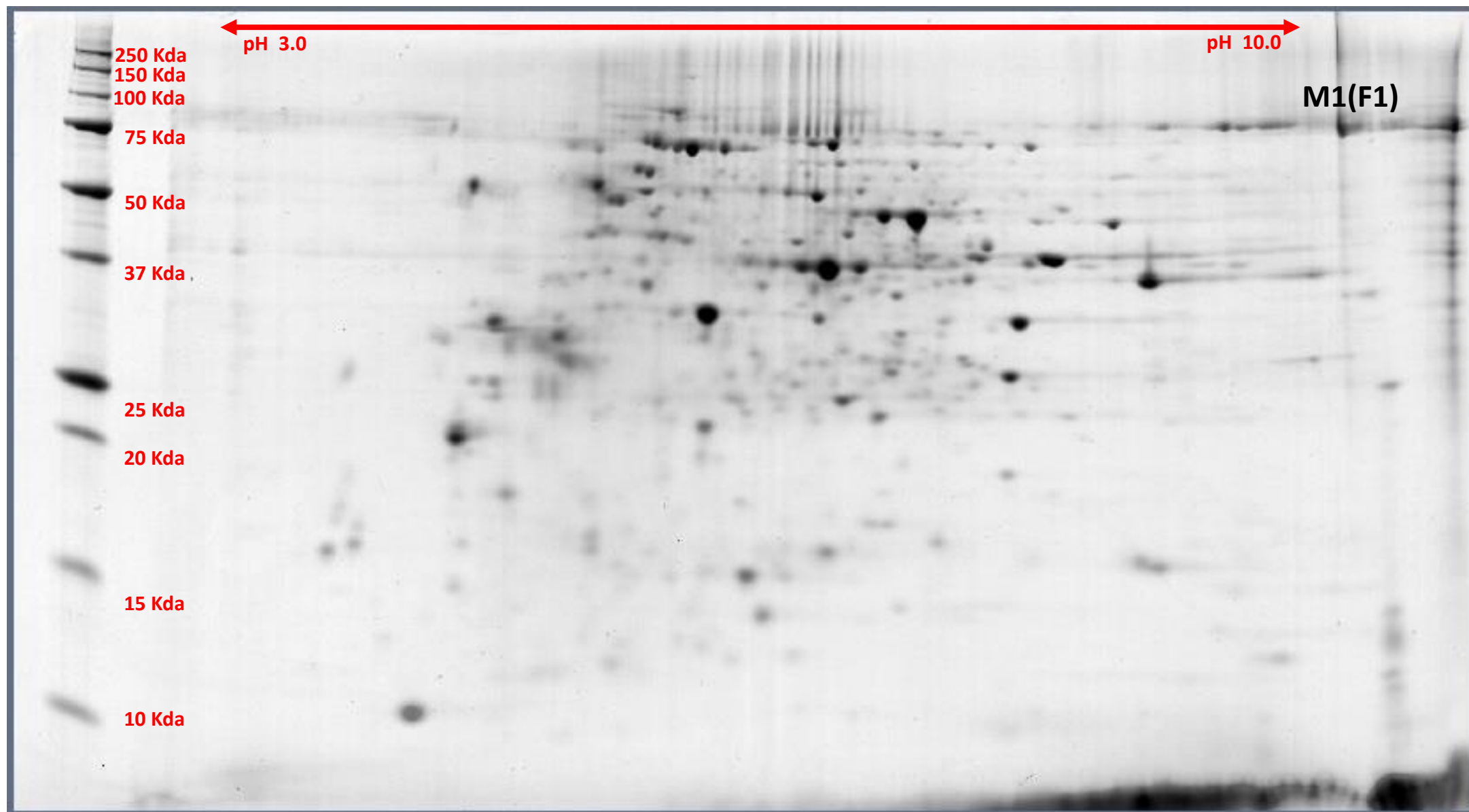

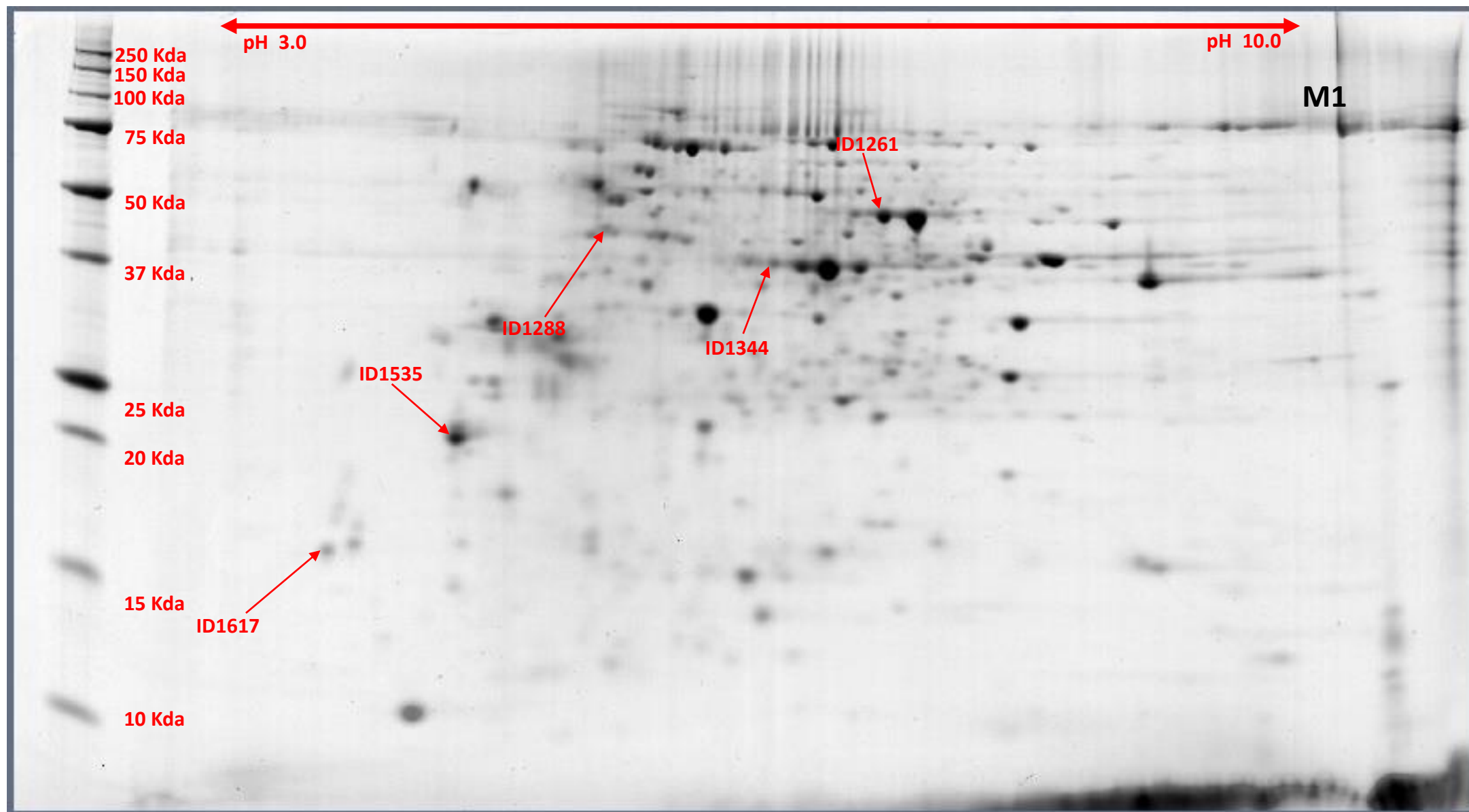

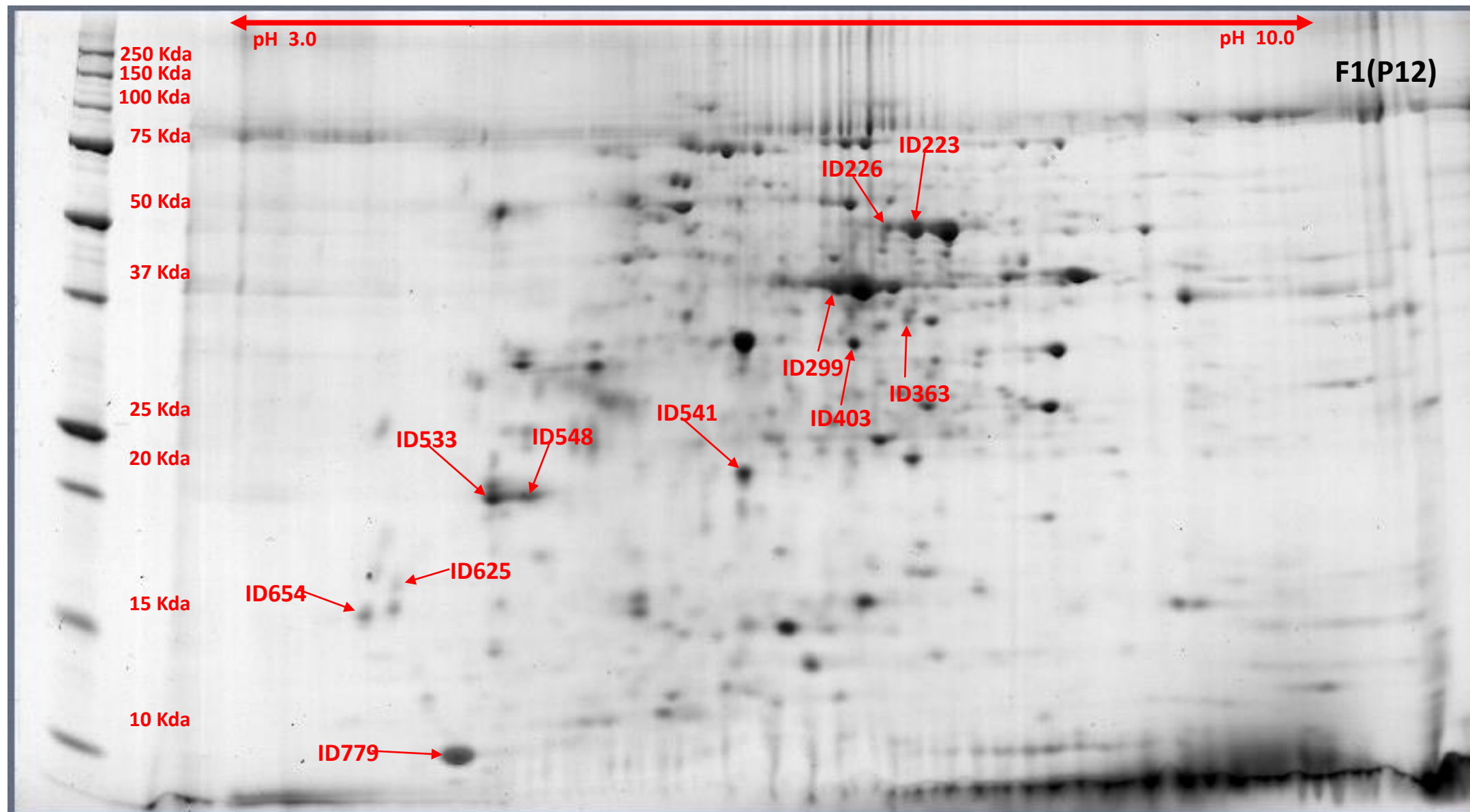

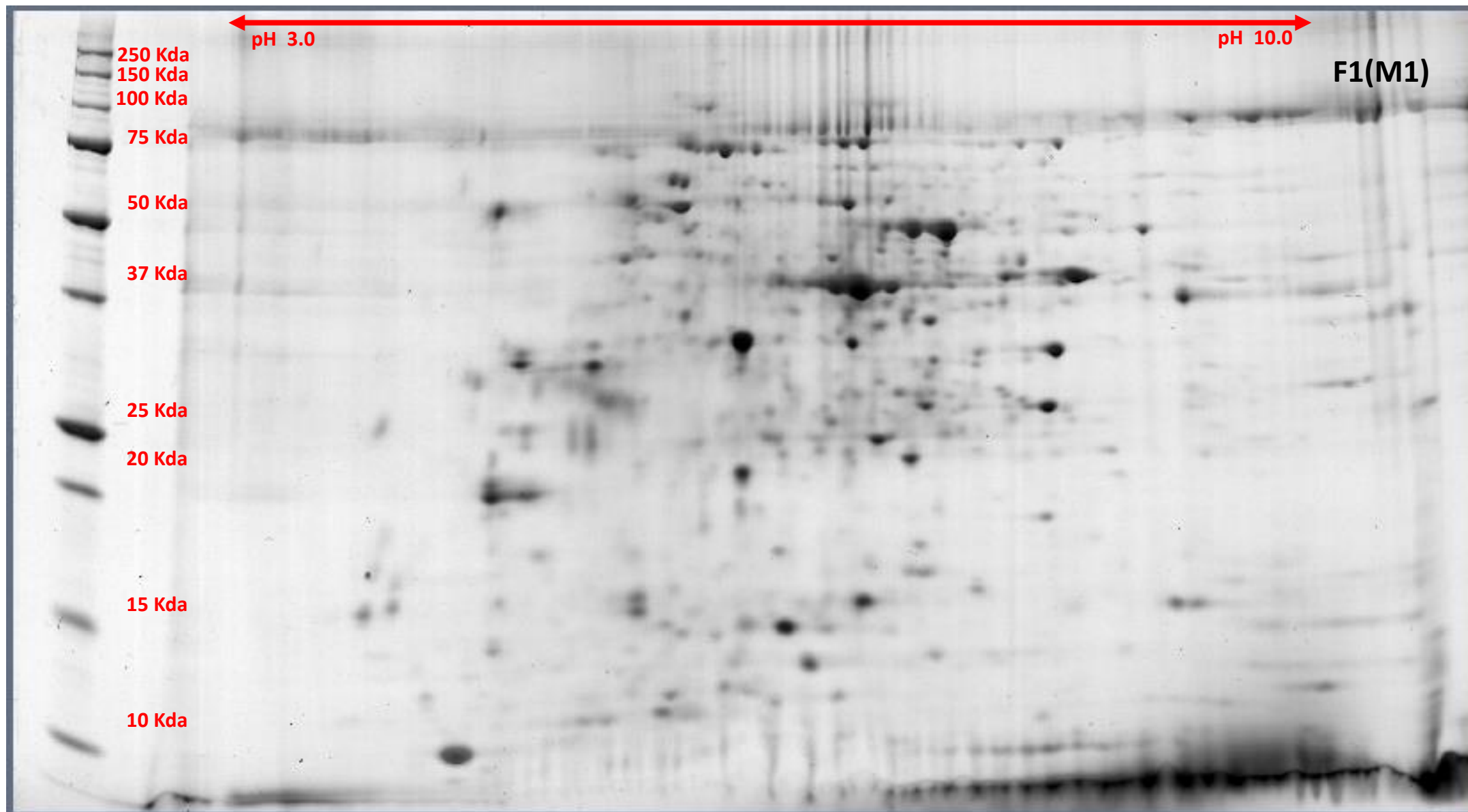

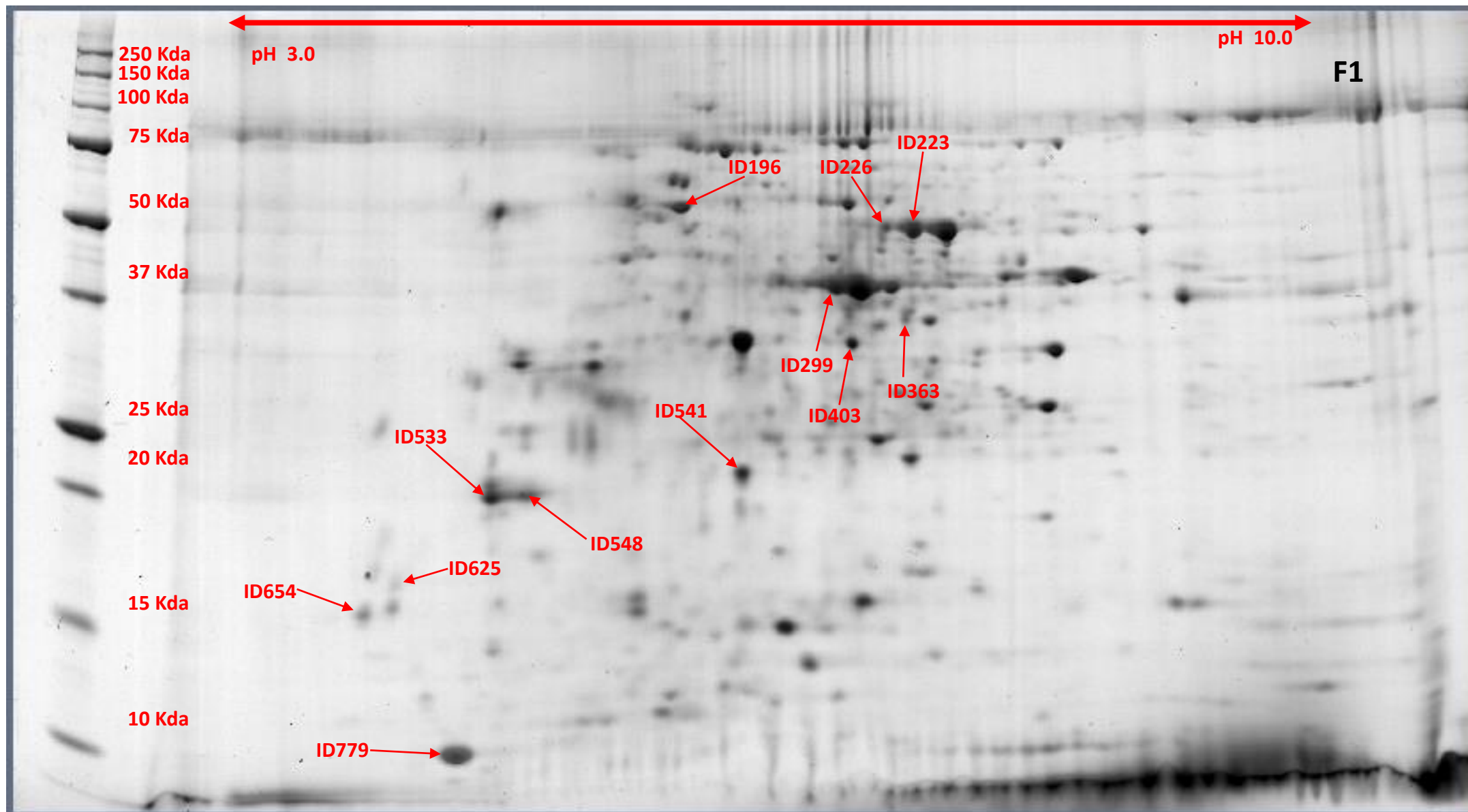

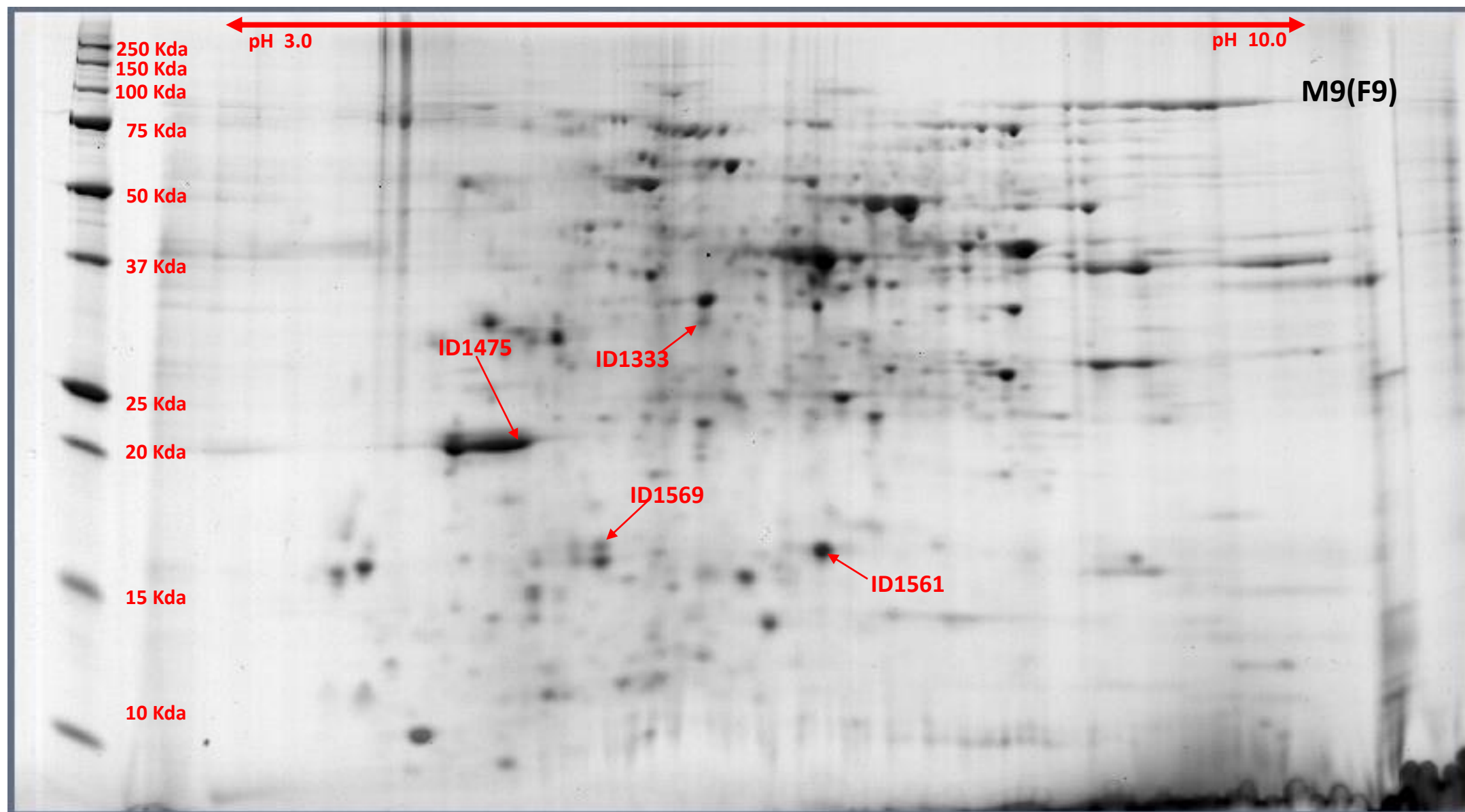

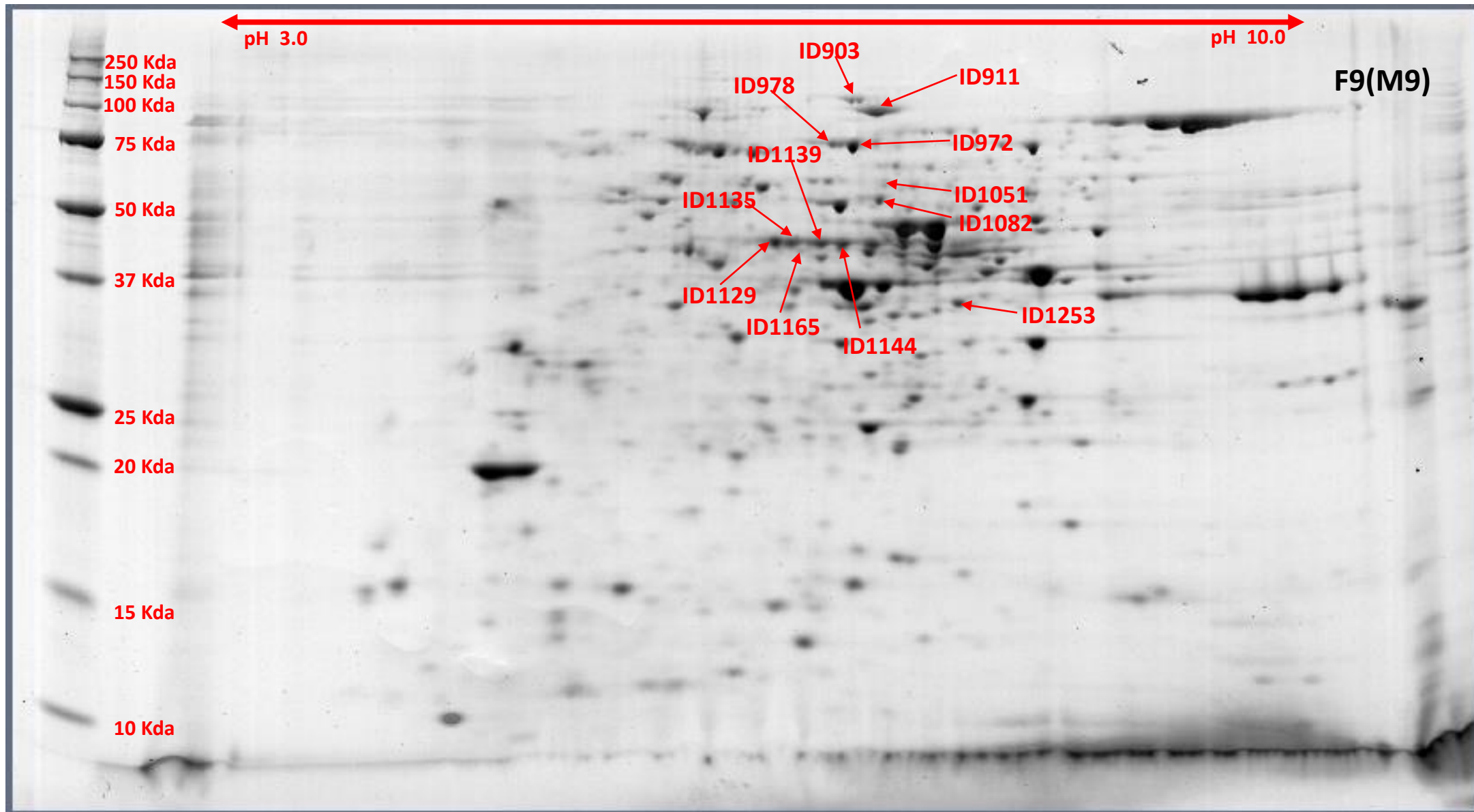

Supplement: Supplementary file 1 [file ijms-19-01996-s001.zip › Supplementary file 3.pdf]
